# Strategies for Controlling Through-Space Charge Transport in Metal-Organic Frameworks via Structural Modifications

**DOI:** 10.3390/nano10122372

**Published:** 2020-11-28

**Authors:** Christian Winkler, Egbert Zojer

**Affiliations:** Institute of Solid State Physics, NAWI Graz, Graz University of Technology, Petersgasse 16, 8010 Graz, Austria; christian.winkler@student.tugraz.at

**Keywords:** metal-organic frameworks, charge transport, through-space pathways

## Abstract

In recent years, charge transport in metal-organic frameworks (MOFs) has shifted into the focus of scientific research. In this context, systems with efficient through-space charge transport pathways resulting from *π*-stacked conjugated linkers are of particular interest. In the current manuscript, we use density functional theory-based simulations to provide a detailed understanding of such MOFs, which, in the present case, are derived from the prototypical Zn_2_(TTFTB) system (with TTFTB^4−^ corresponding to tetrathiafulvalene tetrabenzoate). In particular, we show that factors such as the relative arrangement of neighboring linkers and the details of the structural conformations of the individual building blocks have a profound impact on bandwidths and charge transfer. Considering the helical stacking of individual tetrathiafulvalene (TTF) molecules around a screw axis as the dominant symmetry element in Zn_2_(TTFTB)-derived materials, the focus, here, is primarily on the impact of the relative rotation of neighboring molecules. Not unexpectedly, changing the stacking distance in the helix also plays a distinct role, especially for structures which display large electronic couplings to start with. The presented results provide guidelines for achieving structures with improved electronic couplings. It is, however, also shown that structural defects (especially missing linkers) provide major obstacles to charge transport in the studied, essentially one-dimensional systems. This suggests that especially the sample quality is a decisive factor for ensuring efficient through-space charge transport in MOFs comprising stacked *π*-systems.

## 1. Introduction

Metal-organic frameworks (MOFs) are porous, highly crystalline solids consisting of inorganic nodes connected by organic linkers [1,2,3]. They have been investigated extensively for various applications in fields such as gas storage, [4,5,6] catalysis, [7,8,9], and gas separation [10,11]. Until recently, comparably little attention has been paid to the electronic properties of MOFs, [12] although electrically conductive MOFs can be relevant as active materials for several applications, such as electrocatalysis, [13,14,15,16,17] chemiresistive sensing, [18,19,20,21,22,23] and energy storage [24,25,26]. Therefore, in recent years, interest in controlling and modifying the electronic properties of MOFs has gained considerable attention [12,27,28].

On more fundamental grounds, the electronic (and also the optical) properties of a solid are determined (in a first approximation) by its electronic band structure, where MOFs usually show rather flat bands [27]. This is a consequence of the commonly observed weak hybridization between states localized on the organic linkers and states localized on the inorganic secondary building units. A second reason is the small overlap of the *π-systems* of neighboring organic linkers. This assessment already comprises two strategies for changing the electronic properties of MOFs: one can either focus on improving the bonding between the metal and the ligands (through-bond coupling), or one can try to improve the overlap of the *π-systems* in neighboring linkers (through-space coupling) [12,27,29]. In the present contribution, we will focus on the latter approach, where a large overlap of neighboring *π*-electron systems can result in the formation of bands displaying a large dispersion and generating through-space charge transport pathways.

The impact of the packing motif of organic *π-systems* on intermolecular electronic couplings (expressed via transfer integrals and band dispersions) has been thoroughly studied for organic semiconductors, OSCs. Crucial factors identified for these materials are the stacking distance of neighboring molecules (i.e., neighboring *π*-systems) and the arrangement of neighboring molecules in terms of relative displacements and rotations [30,31,32,33,34,35,36,37]. All these structural changes lead to changes in the orbital overlap between consecutive molecules, which, in turn, change the intermolecular electronic couplings. For example, considering dimers of acenes (or quinacridone) and shifting the molecules relative to each other along their long molecular axis, one finds that the transfer integrals oscillate as a function of the displacement [30,31,32,33,34,36,38]. The influence of relative rotations of molecular dimers has been investigated primarily in the context of discotic liquid crystals. There, it has been found that the transfer integral varies as a function of the rotation angle [35,37,39,40,41,42]. Importantly, independent of whether neighboring molecules are shifted or rotated relative to each other, what counts for the intermolecular electronic coupling (transfer integral) is the overlap of the frontier orbitals. This overlap is determined by the orbitals’ shape and symmetry. In this context, it has been suggested that when organic semiconductors can assemble without pronounced sterical constraints, exchange repulsion acts as an intrinsic driving force, favoring molecular arrangements with particularly small electronic couplings [32,33]. Therefore, developing design strategies “extrinsically” enforcing favorable molecular arrangements have shifted into the focus of current research [32,33,43,44,45,46]. Here, MOFs are of particular appeal, as the framework structure offers an additional level of control over the stacking sequence of neighboring molecules, which goes far beyond what is typically achievable in organic semiconductors. Similar to OSCs, it has been predicted also for layered MOFs that their electronic band structure depends on the proximity [47] of the layers as well as on interlayer displacements [48,49,50]. For MOFs comprising 3D networks, such structure-to-property relations for charge transport are, however, hardly developed.

Material-wise, particularly promising MOFs showing through-space charge-transport pathways are frameworks consisting of ligands based on tetrathiafulvalene (TTF) [12]. Especially for a subgroup of these MOFs in which the TTF units form helical stacks with comparably close *π*-stacking one observes relatively large electrical conductivities [12,51]. For such systems, it has also been shown that reducing the S…S stacking distance within the TTF stacks results in significantly increased conductivities [52,53,54].

In this work, we will apply dispersion-corrected density functional theory (DFT) calculations to show, how the electronic coupling in such TTF-based MOFs can be controlled by additional structural parameters, such as the relative rotation or slip of neighboring TTF units. The goal of these calculations is to identify stacking motives that maximize through-space charge-carrier mobilities. Moreover, we will address the impact of defects such as missing linkers, pair formation, and shifted molecules. 

### 1.1. Systems of Interest

**Figure 1:**
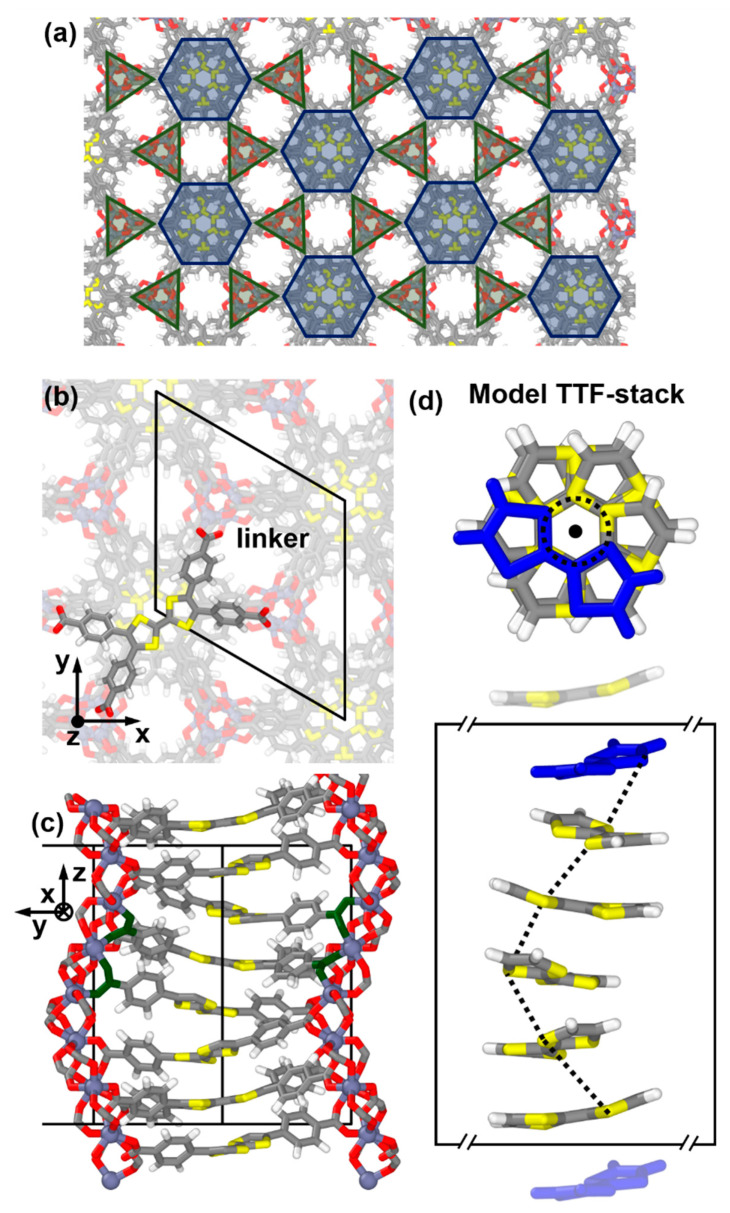
Structure of Zn_2_(TTFTB) and the constructed tetrathiafulvalene (TTF) model system. Panel (**a**) shows the connectivity between linkers (blue hexagons) and nodes (green triangles) within the Zn_2_(TTFTB) metal-organic framework (MOF). Panels (**b**,**c**) contain a more detailed illustration structure of the Zn_2_(TTFTB) MOF (top and side view). The linker is highlighted in (**b**), and in panel (**c**), the carboxyl groups of neighboring linkers are colored in green to indicate how linkers and metal nodes are connected on an atomistic level. The unit cell of the MOF is represented by thick black lines. Panel (**d**) contain the top and side views of the model system used for describing the one-dimensional charge transport in these materials. The top TTF molecule in the model system is marked in blue, and the rotation axis is indicated by the black dot in the center of the top structure in panel (**d**). The dashed black line in the model system indicates the rotation of the molecules around the screw axis. The periodic boundary conditions are indicated by the frame around the repeat unit of the TTF model system. C—grey, H—white, S—yellow, Zn—purple, O—red.

The starting point for this study is the stacking of the TTF cores of Zn_2_(TTFTB) [51], shown in Figure 1. The linkers (TTFTB^4−^ = tetrathiafulvalene tetrabenzoate) and the metal nodes (forming Zn_2_(TTFTB)) crystallize in the P6_5_ space group with a hexagonal unit cell (a = b= 19.293 Å and c = 20.838 Å). This results in helical TTF stacks (six TTF molecules per unit cell), where neighboring TTF molecules are rotated by 60° relative to each other and translated by 3.473 Å in stacking direction (see Figure 1). The stacks themselves are arranged in a hexagonal pattern and connected by the nodes, as illustrated in Figure 1a. Notably, the 6_5_ screw axis is offset from the central ethylene unit of the TTF cores (see Figure 1b,c). This induces an additional shift of neighboring molecules relative to each other, which is perpendicular to the screw axis [51]. As a consequence, the centers of the TTF molecules are arranged on a helix, whose projection onto the x,y-plane (the plane perpendicular to the stacking direction, z) becomes a circle with a radius r of ~1.6 Å. This stacking motif of the TTF cores is determined by the arrangement of the Zn nodes and the carboxylic acid groups. The MOF structure discussed in the main manuscript contains neither solvent molecules nor water molecules (i.e., the MOF is desolvated and dehydrated). For comparison, a MOF with water molecules coordinating to the Zn atoms was also calculated. The water molecules cause only very minor changes in the atomic coordinates and the electronic structure (see Supplementary Materials). 

For analyzing the impact of structural changes on the electronic properties of the TTF stacks, we first constructed helical model TTF and TTFTB stacks consisting of molecules exhibiting the same geometry and stacking motif as in the MOF. These stacks were then arranged in the same pattern as in the MOF, as shown in Figure 1a. 

For generating TTF stacks with different numbers of molecules in the unit cell, N, we replicated individual molecules (in the geometries adopted in the stacks), rotated them around the central screw axis by angles of 360°/N, and arranged them at distances of 3.473 Å. Laterally, these stacks were then, again, arranged consistent with the experimental structure of Zn_2_(TTFTB), while the unit cell in the stacking direction was set to N*3.473 Å. To verify the construction procedure, we compared the electronic structure of the N = 6 TTF model stack to the system extracted directly from the MOF structure, observing only negligible differences (see Table 1 in Section 3.2). A detailed description of the construction of the parent TTF stacks and the model systems with modified rotation angles can be found in the Supplementary Materials. 

Additionally, molecular dimers were designed in analogy to the construction described in the previous paragraph. As these dimers were simulated using open boundary conditions, any value could be chosen for the rotation angle around the off-center screw axis, allowing us to generate smooth evolutions of the dimer electronic couplings with rotation angle.

To investigate the impact of chemical modifications, we also considered Cd_2_(TTFTB), which has been reported to be isostructural to Zn_2_(TTFTB) but shows a higher electrical conductivity [53]. Moreover, we replaced TTF with tetraselenafulvalene (TSF, C_6_H_4_Se_4_, yielding Zn_2_(TSFTB)) to test the extent to which the increased p_z_-orbital overlap for Se would result in a larger valence bandwidth.

### 1.2. Describing Through-Space Charge Transport in Pristine MOFs

Before considering the electronic band structure of Zn_2_(TTFTB) and how it is affected by changes in the structure of the TTF stack, it is useful to realize that through-space charge transport in porous materials is strongly reminiscent of the situation in (one-dimensional) organic semiconductors, [31,55,56,57] for which various models for describing charge transport have been proposed over the past few decades. These models comprise fully coherent band transport and incoherent hopping transport as limiting cases and also include more recent developments, such as the highly successful dynamic disorder model [58,59,60,61,62]. For all these models, the intermolecular electronic couplings between neighboring molecules are essential parameters [31,55,56,57]. These electronic couplings are typically expressed via transfer integrals t, which enter linearly (quadratically) into the expressions for band (hopping) mobilities [31,57]. Such transfer integrals can, for example, be extracted from studying suitably arranged dimers [31,34,35,36,37,63] or from fitting tight-binding models to electronic band structures [64]. In fact, within a tight-binding picture, the magnitudes of the transfer integrals can be intimately related to the widths of the frontier bands. This suggests that general trends for the dependence of charge-carrier mobilities on structural parameters can be gained from calculating electronic band structures, even in cases in which band transport is not the dominant mechanism. Therefore, in the following, we will primarily analyze such band structures calculated by dispersion corrected density functional theory (with the discussion primarily based on band widths and the derived transfer integrals). We acknowledge that in this way, the role of the material’s phonon properties (such as the occurrence of “killer phonon” modes) [65] is not revealed. Nevertheless, the analysis provides crucial insights into the structure-to-property relationships for the electronic MOF properties which determine through-space charge transport.

## 2. Methods 

For investigating the structural and electronic properties of the MOFs, the periodic model systems, and the molecular dimers, we employed the dispersion-corrected density functional theory, DFT, which, in a recent review, was highlighted as a viable method for gaining an in-depth understanding of the electronic structure of MOFs [66]. The simulations were performed with the FHI-aims code (version 190906, Berlin, Germany) [67]. Exchange and correlation were treated by the PBE functional [68,69], and the Tkatchenko–Scheffler [70] scheme was used as an a posteriori van der Waals correction. We employed the default tight basis sets of FHI-aims for periodic and molecular simulations. Further details on the employed basis functions are provided in the Supplementary Materials. For Zn_2_(TTFTB), the electronic band structure was also calculated with the hybrid functional HSE06 [71,72] to ensure that the introduction of Hartree–Fock exchange has a negligible influence on the valence bandwidth as the primary quantity of interest for the present study.

During the geometry optimizations, all atomic positions were relaxed until the largest remaining force component on any atom was smaller than 0.01 eV/Å. For all MOF systems, a 3 × 3 × 3 k-point grid was used for sampling reciprocal space during the geometry relaxations. During the electronic structure calculations, a 4 × 4 × 4 k-point grid was employed. For both grids, the total energy was converged to within less than 1 meV. The smaller grid in the more time-consuming geometry relaxations was used to speed up the calculations. For the periodic stacks, we used a 1 × 1 × 12 k-point grid, which is already converged, even for the smallest system (with the largest reciprocal space vector along the stacking direction). The effective masses were calculated from the (inverse) curvature of the band structure at the top of the valence band in the (001) direction to describe transport in the TTF stacking direction. Technically, the band curvature was determined by fitting a cosine function to the dispersion relation E(k), including the 10 k-points closest to the valence band maximum, with a spacing between neighboring k-points of 0.005 Å^−1^. A cosine function was chosen for the fit to be consistent with the best-suited tight-binding band model for the systems studied here (see below). The structures of the MOFs and the molecular systems were visualized using Ovito (version 3.2.1) [73] and the molecular orbitals were rendered using Avogadro (version 1.2.0) [74].

## 3. Results and Discussion

### 3.1. Electronic Structure of Zn_2_(TTFTB) and the Extracted Model Stack

**Figure 2:**
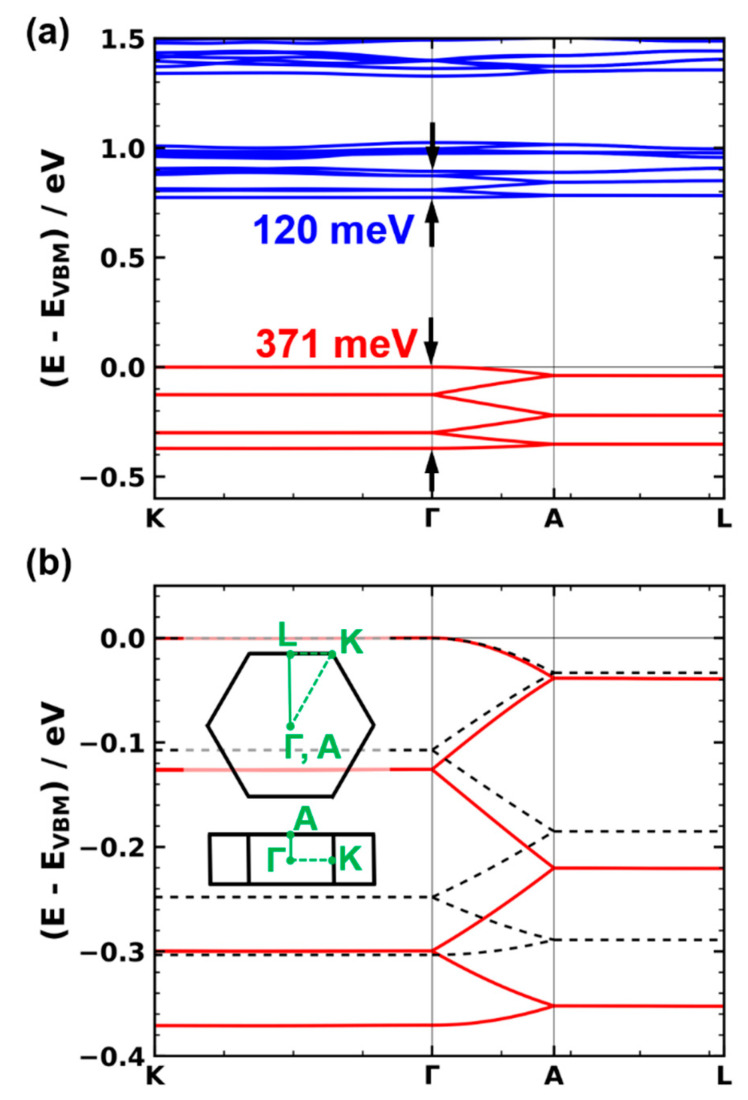
Electronic structure of the Zn_2_(TTFTB) MOF and the corresponding TTF model stack: (**a**) electronic band structure of Zn_2_(TTFTB) along the selected high-symmetry directions. The energy scale is aligned to the valence band maximum. (**b**) Zoom into the valence band of Zn_2_(TTFTB) (solid red line) and of the TTF model stack (dashed black line). The first Brillouin zone together with the relevant directions in k-space are shown as an inset.

As a first step, we analyzed the electronic structure of Zn_2_(TTFTB), for which the frontier bands are shown in Figure 2a. Figure 2b contains a zoom into the valence band region. In the following, we will be primarily concerned with bands in the ΓA direction, as this corresponds to the stacking direction of the TTF molecules. Moreover, the valence and conduction bands are flat in directions perpendicular to ΓA (with bandwidths around 1 meV in AL and even less in ΓK directions). This indicates that there is virtually no electronic coupling between individual TTF stacks within the MOF, resulting in essentially one-dimensional charge transport in Zn_2_(TTFTB). This is supported by measurements by Sun et al., who observed that the electrical conductivity in the direction of the stacks is 2–3 orders of magnitude larger than perpendicular to them [75]. 

As far as the ΓA direction is concerned, one can identify a six-times backfolded band, which is particularly well resolved for the valence band in Figure 2b. This backfolding is related to the crystallographic unit cell (determining the shape and size of the first Brillouin zone). It contains six TTF-based linkers as translational repeat units, whose repetition yields the infinitely extended TTF stack. What counts from an electronic point of view is, however, not only the translational symmetry but also the screw axis in the middle of the TTF stack (see Figure 1). With respect to that screw axis, each TTF molecule has an identical electronic environment. Thus, one can view a single TTF molecule as the “electronic” repeat unit of the TTF stack in Zn_2_(TTFTB). This is supported by the observation that for the perfectly symmetric structure, no band gaps open for the backfolded bands at the Γ and A points. In passing, we note that this situation changes when defects disturb the perfect symmetry, as will be discussed in Section 3.4.

**Figure 3:**
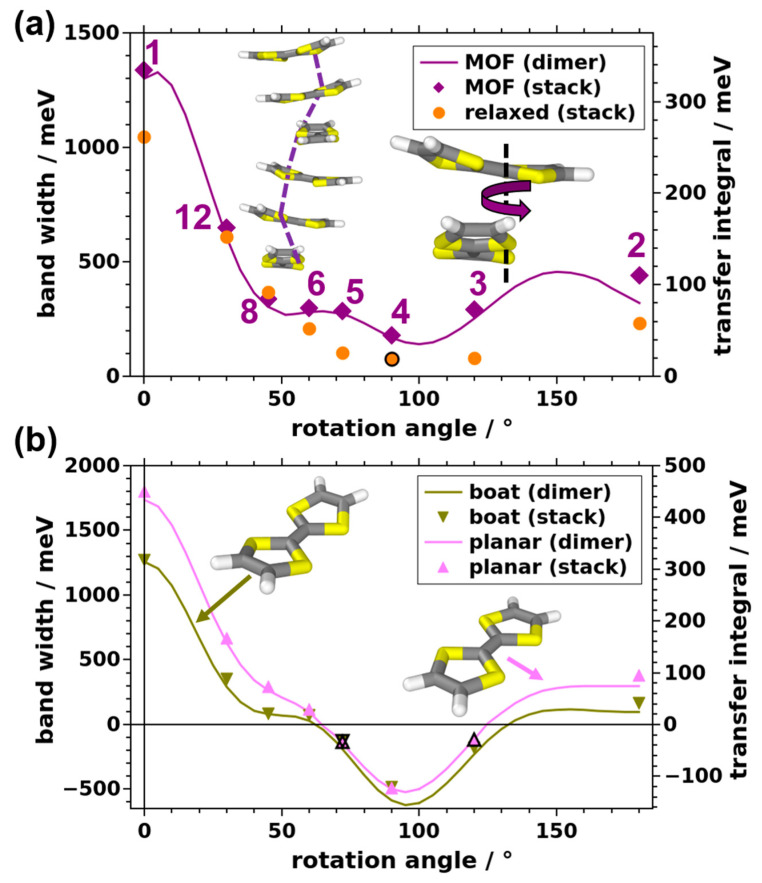
Evolution of the width of the valence band in the (001) direction and of the transfer integrals between neighboring TTF molecules in the stacking direction as a function of the rotation angle between neighboring molecules. Panel (**a**) shows the situation for stacks and dimers with molecular geometries taken from Zn_2_(TTFTB) (purple diamonds and line) and for stacks with optimized geometries (orange circles; for details, see main text). In panel (**b**), the results for fully gas-phase optimized (dark yellow down-facing triangles and line) and for planar molecules (light magenta up-facing triangles and line) are shown. Symbols denote data points calculated for infinitely extended TTF stacks, where the rotation angles are set by varying the number of TTF molecules in each unit cell (see numbers in panel (**a**)). The solid lines have been calculated for dimers with rotation angles varied in steps of 5° (individual data points not shown). In panel (**b**), bandwidths are set to negative values whenever the signs of the dimer-calculated transfer integrals are also negative. Points marked with a black frame comprise band structures deviating from a simple 1D tight-binding system and are discussed in the Supplementary Materials.

As a consequence of a single TTF molecule serving as an “electronic” repeat unit, the electronic bands in the ΓA direction can be conveniently described by a tight-binding model with a single molecule per unit cell. These considerations imply that for judging the electronic coupling between neighboring TTF molecules, one needs to consider the width of the entire, six-times backfolded band, as indicated by the arrows in Figure 2a. Based on the 1D nearest-neighbor tight-binding model mentioned above, the total band width of the six-times backfolded band then corresponds to 4 × t (with t representing the intermolecular transfer integral in stacking direction). This extraction of t from the band structure is confirmed by the data shown in Figure 3, where the dimer-derived bandwidths are compared to the results for the actual TTF stacks. Additional validation data are contained in the Supplementary Materials. Conversely, the width of the valence band between Γ and A is a measure for the electronic coupling between adjacent groups of six TTF units (i.e., between the entirety of the TTF molecules in adjacent unit cells). 

On more quantitative grounds, the valence bandwidth, VBW, for the backfolded band amounts to 371 meV in the PBE calculations (400 meV when using the HSE06 functional), as indicated by the arrows in Figure 2a. This is significantly larger than the width of the conduction band, which is 120 meV. This finding suggests that Zn_2_(TTFTB) is preferentially a hole conductor [53], which is in line with the notion of organosulfur compounds, such as TTF, being good electron donors [76,77].

An analysis of the contributions of the different parts of the MOF to the valence and conduction bands suggests that especially hole transport (which is particularly relevant for TTF-based systems; see above) should be described well by the model TTF stack. This notion is confirmed by the comparison of the valence band structure of the actual Zn_2_(TTFTB) MOF (solid line) and the band structure of the model TTF stack in Figure 2b; qualitatively, the two band structures are the same. The only apparent difference is a somewhat smaller bandwidth of 303 meV in the model system (which amounts to ~82% of the bandwidth of the actual MOF). This leads to a comparably small change in the effective mass at the valence band maximum (VBM) from 2.05 to 2.40 m_e_ (with m_e_ being the mass of a free electron). We attribute this difference to the overlap of the *π*-orbitals of neighboring phenylenes in the H_4_TTFTB linkers in the actual MOF, which is not captured by the model system (see systems TTF and TTFTB in Table 1 and further details in the Supplementary Materials). These quantitative differences are, however, rather small compared to the effects discussed below, rendering the TTF stack a useful model system.

### 3.2. Dependence of Bandwidth and Transfer Integral on the Relative Rotation of Consecutive TTF Units

With a reliable model system at hand, we can now turn to studying the impact of changes in the structure of the TTF stacks on the electronic coupling. It has been shown, for a variety of molecular dimers, that the relative rotation of neighboring molecules has a tremendous effect on intermolecular electronic couplings [35,37,39,40,41,42]. As this effect is a consequence of changes in the orbital overlap upon rotation, one can expect similar effects for the TTF stacks considered in this work. Following the construction procedure for TTF stacks described in Section 1.1 and in the Supplementary Materials, it is apparent that the number of stacked molecules in the unit cell determines their relative rotation. Thus, we consider unit cells containing 1, 2, 3, 4, 5, 6, 8, and 12 molecules (corresponding to rotations of 0°, 180°, 120°, 90°, 72°, 60°, 45°, and 30°, respectively).

The resulting band structures are shown in the Supplementary Materials. They reveal that the cofacial arrangement with one repeat unit exhibits the largest valence band width of 1337 meV, corresponding to a transfer integral between neighboring molecules of 334 meV (see data points in Figure 3a and values in Table 1). The bandwidth decreases by a factor of nearly three to 447 meV when considering the system with two molecules per unit cell (N = 2, or a relative rotation between consecutive TTF molecules of 180°). The bandwidth further decreases for three TTF molecules (120° rotation) and reaches a minimum of 180 meV (a transfer integral of 45 meV) for the system containing four molecules in the unit cell (see Figure 3a). Upon further increasing the number of repeat units, the bandwidth again increases slightly (to between 235 and 337 meV for N = 5, 6, and 8). A steep increase is then observed for 12 molecules per unit cell (i.e., for a relative rotation angle of 30°). Here, a valence bandwidth of 650 meV means a doubling compared to the reference system with N = 6, which mimics the situation in the actual Zn_2_(TTFTB) MOF. Concomitantly, the effective mass of the holes increases from 0.93 m_e_ for N = 1 to 2.48 m_e_ for the reference system with N = 6 and then drops again to 1.84 m_e_ for N = 12. These considerations show that changing the relative twist between consecutive molecules, indeed, has a profound impact on the electronic coupling in the TTF stack and that the situation in Zn_2_(TTFTB) is far from ideal for hole transport.

For obtaining values at intermediate rotation angles we considered model dimers, extracting transfer integrals via the fragment orbital method [78]. The valence bandwidth for one molecule as “electronic repeat unit” is then obtained as W = 4 × t employing a 1D tight-binding model. At rotation angles at which data for actual stacks and from dimers are available, one typically observes an excellent agreement. This suggests that dimer calculations can, indeed, be used as an efficient tool for predicting and explaining general trends.

As a next step, we discuss the role of the conformation of the molecules within the stack. First, we fully relaxed the geometries of the molecules in the stacks, fixing only the positions of the central C=C atoms to maintain the overall structure. This yields an evolution of the valence bandwidths comparable to that of the TTF stacks with molecules in MOF geometry (see above), although the bandwidths are consistently smaller in the relaxed case (with the exception of the N = 8 system; see Figure 3a). The reduction in the bandwidth is particularly pronounced for N between 2 and 5, such that the overall variation between the largest and the smallest bandwidths amounts to a factor of almost 14.

For the relaxed stacks, it is also sensible to compare the total energies of the systems. Interestingly, for N between 3 and 12 these are within 35 meV per molecule (i.e., only somewhat larger than k_B_T; see Supplementary Materials). This occurs despite variations in the bandwidths (transfer integrals) by a factor of 8. Only for N = 1 and 2, the total energy increases by 232 and 121 meV, respectively. This suggests that from a TTF-stacking point of view, there is no strong driving force preventing structures with comparably large bandwidths (such as for N = 12), which is in sharp contrast to observations for various molecular crystals [32,33].

Notably, in the stacks discussed so far (fully optimized or built from molecules in MOF conformation), the TTF molecules are slightly tilted around their long and short molecular axes. To assess the role of those tilts, we also studied two model systems in which such tilts do not occur, starting from a gas-phase optimized TTF molecule either in boat conformation (actual minimum structure) or forced to be planar. In the stacks, these molecules are then aligned such that all S atoms of each molecule are in a plane perpendicular to the screw axis. The infinitely extended stacks are then constructed following the procedure described in Section 1.1 and in the Supplementary Materials. The results for these stacks are complemented by calculations for corresponding molecular dimers. As shown in Figure 3b, the obtained data, at first glance, appear to directly correlate to the data for the MOF-derived structure (purple diamonds and line in Figure 3a). A closer inspection, however, reveals that there is a fundamental difference: the signs of the dimer transfer integrals come out negative for rotation angles Θ between ~65° and ~125° (where, for the sake of consistency, we also plot the bandwidths with a negative sign in that range of rotation angles). The zero-crossing of transfer integrals and bandwidths has a profound impact on charge transport properties. As for systems like the present one primarily the absolute value of the bandwidth counts, for the cases shown in Figure 3b the carrier mobility in stacking direction is expected to be close to a local maximum for the N = 4 system (rather than close to the global minimum, as for the systems shown in Figure 3a). Conversely, the valence bands become completely flat for rotation angles around ~65° and ~125°, implying a vanishingly small carrier mobility in for these angles.

**Figure 4:**
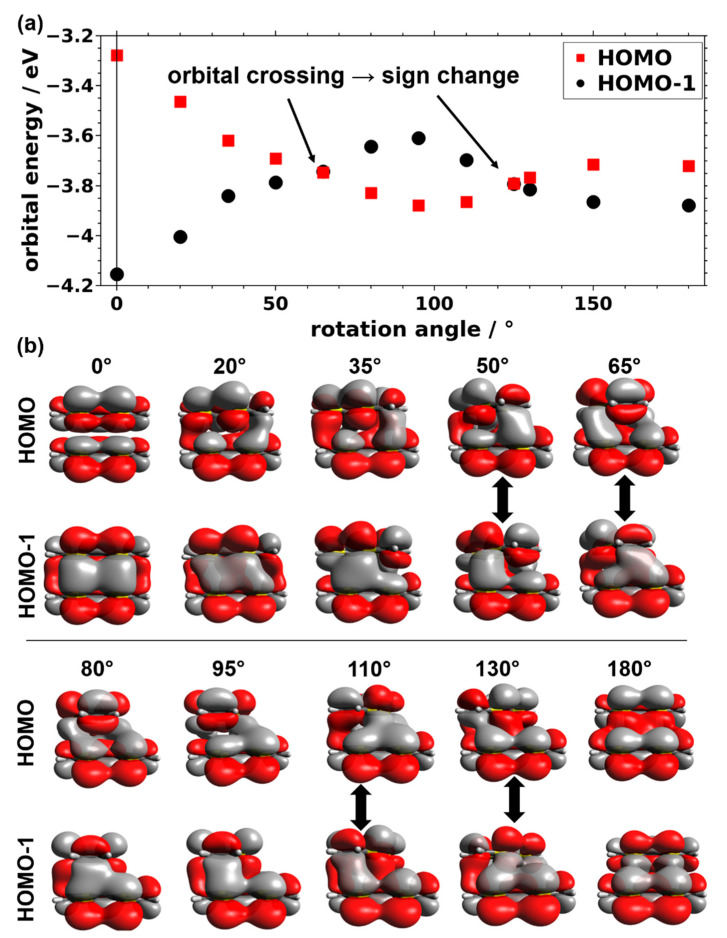
(**a**) Orbital energies of the planar TTF dimer as a function of the rotation angle and (**b**) corresponding plots showing the respective molecular orbitals. The dimers at the angles between which the two frontier orbitals change their order are highlighted by thick, vertical black arrows in panel (**b**).

The evolution of the transfer integrals with rotation angle (including the zero-crossing) can be explained by the shapes of the involved molecular orbitals. This is most straightforwardly seen for the bonding and antibonding linear combinations of the highest occupied molecular orbitals (HOMOs) of the two TTF molecules in the dimer calculations. They can be derived from linear combinations of the HOMOs of individual TTF molecules, and (for centrosymmetric systems) their splitting determines the magnitude of the transfer integral [31]. The evolutions of the orbital shapes and orbital energies with rotation angle are exemplarily shown in Figure 4 for the dimers consisting of planar molecules.

For the cofacial arrangement of the molecules, the antisymmetric linear combination of the TTF HOMOs is lowest in energy and the symmetric linear combination is highest (Figure 4b, 0°). This is exactly what one would expect considering the fully bonding nature of the hybrid orbital in the antisymmetric case (non-zero value of the wavefunction between molecules or even a local maximum) and its fully antibonding nature in the symmetric case (vanishing wavefunction between the molecules). Upon increasing the rotation angle, the HOMO-1 becomes increasingly destabilized and the HOMO gets stabilized, which reduces their energetic splitting and, concomitantly the associated transfer integral. This can be understood by the appearance of antibonding contributions for the antisymmetric and bonding contributions for the symmetric linear combinations. At a rotation angle of 65°, both linear combinations display nearly equal amounts of bonding and antibonding regions. Consequently, the two orbitals are essentially isoenergetic, resulting in a vanishing transfer integral. Upon further increasing the rotation angle, the nature of the HOMO and HOMO-1 is switched, resulting in a change of the sign of the transfer integral. The stabilization of the originally antisymmetric linear combination of the molecular orbitals is maximized at a rotation angle of 95° and the trend is reversed for systems with larger rotations. In passing, we note that the reason for the much smaller HOMO-to-HOMO-1 splitting at 180° compared to the cofacial situation (i.e., 0°) is the reduced spatial overlap of the molecules following from the screw axis not coinciding with the center of the TTF molecules (see Figure 1).

Similar trends are observed for the other three molecular conformations. The lack of a zero-crossing of the bandwidths and transfer integrals for the MOF-derived and optimized geometries (i.e., the systems shown in Figure 3a) arises from the fact that due to the twisting of the molecules around the long and short molecular axes, certain regions of neighboring molecules get particularly close. This strongly amplifies the contributions of these regions to the orbital energies, such that the cancellation effects discussed above do not occur any more.

To conclude this section, it should be noted that, of course, also the location of the screw axis relative to the center of the TTF molecules impacts the wavefunction overlap, as discussed in detail in the Supplementary Materials. In short, the resulting overall situation is similar to the cases discussed above, although in that case, there is no zero-crossing of the transfer integral for the planar molecular conformation.

### 3.3. Impact of the Intermolecular Distance and of Chemical Modifications on the Bandwidth

**Figure 5:**
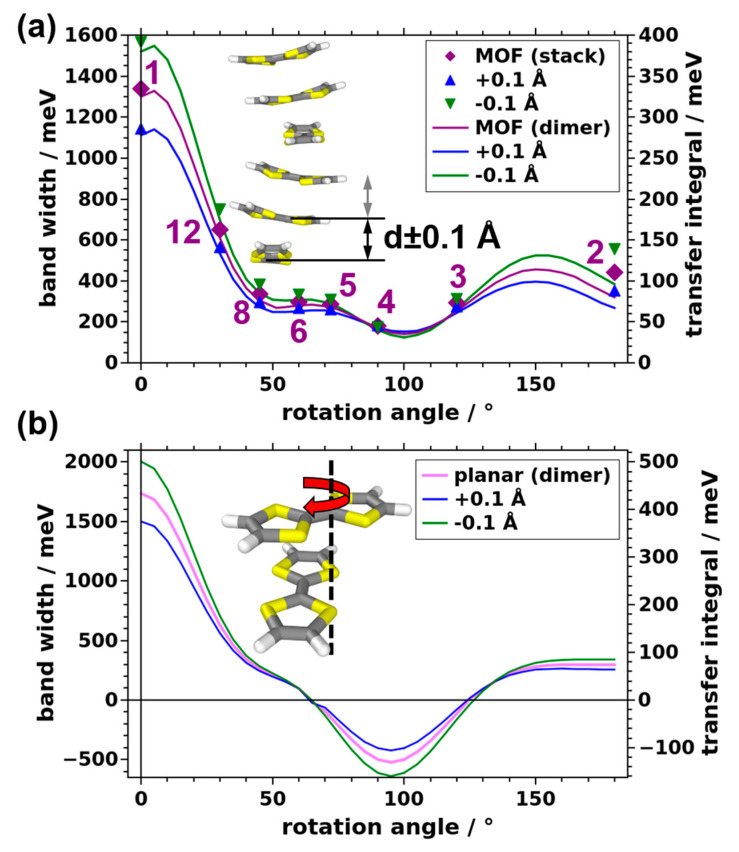
Evolution of the valence bandwidth and the transfer integral with the rotation angle as a function of the stacking distance. (**a**) Results obtained for TTF stacks and corresponding dimers based on the MOF geometry. (**b**) Dimer results based on the planar TTF conformation.

Another structural parameter that is expected to change the intermolecular electronic coupling is the distance between neighboring TTF molecules. In fact, for layered MOFs, a profound impact of the layer proximity on the electronic band structure has been predicted [47]. For periodic stacks, such as the ones studied here, the distance between neighboring TTF molecules can simply be changed by modifying the unit cell length in the stacking direction. The impact of changing the stacking distance by ±0.1 Å per molecule is shown in Figure 5a for molecules adopting the same conformation as in the MOF and in Figure 5b for planar molecules. Not unexpectedly, the bandwidth typically increases upon decreasing the inter-molecular distance and vice versa. The data in Figure 5 also show that, typically, the absolute change in bandwidths and transfer integrals with stacking distance is more pronounced for situations in which these quantities are already large to start with. This can be rationalized based on the discussion of Figure 4 in the previous section; in cases in which bonding and antibonding contributions for certain hybrid orbitals largely cancel, the situation is not fundamentally modified upon changing the stacking distance. Conversely, when hybrid orbitals are either fully antibonding or fully bonding (as in the case of the cofacial dimer), changing the stacking distance has a maximal impact.

Notably, for the N = 6 stack, which directly mimics the stacking of the TTF molecules in the actual Zn_2_(TTFTB) MOF, the increase in the valence bandwidth for a reduction in the stacking distance by 0.1 Å amounts to only 33 meV (~11%). This is, insofar, relevant, as it has been reported that changing the stacking distance for TTFTB-based MOFs results in massive changes in the measured electrical conductivities [53]. Especially when replacing the Zn^2+^ cations in the synthesis with Cd^2+^, an increase in the electrical conductivity by two orders of magnitude has been observed. Originally, this was attributed to the lowered S…S distances for neighboring TTF units, which decreased by 0.103 Å [53]. Such a massive change in conductivities, however, cannot be explained by the trends discussed above. This raises the question of whether there are relevant structural changes between the Zn_2_(TTFTB) and Cd_2_(TTFTB) MOFs beyond a change in the stacking distance. Therefore, we compared the full electronic band structures of Zn_2_(TTFTB) and Cd_2_(TTFTB) (see Supplementary Materials), but also, in this case, the changes in bandwidths and effective masses for the valence band are comparably minor, as summarized in Table 1. In fact, the valence bandwidth is even smaller in Cd_2_(TTFTB) than in Zn_2_(TTFTB).

A different approach for increasing the valence bandwidth could be to increase the orbital overlap by exchanging TTF with tetraselenafulvalene (C_6_H_4_Se_4_). In our calculations, this results in an increase in the valence bandwidth by a factor of almost two (from 371 to 641 meV). Considering that we observe only minor structural changes between the S-based Zn_2_(TTFTB) and the Se-based Zn_2_(TSFTB), we attribute that to the larger spatial extent of the p_z_-orbitals of Se, which result in an increased wavefunction overlap. Nevertheless, such chemical modifications also do not change hole mobilities by orders of magnitude.

An additional factor that would influence the mobility of holes would be changes in the vibrational properties of the MOF, which impact charge transport through dynamic disorder effects [57,58,65]. Such effects are not explicitly considered here, but it is hard to imagine that they could be responsible for the orders of magnitude changes in transport properties between Zn_2_(TTFTB) and Cd_2_(TTFTB).

There could, however, be several other explanations for the above-mentioned differences. First, one has to keep in mind that in [53], the authors did not report carrier mobilities but electrical conductivities (which, to date, is still common for metal-organic frameworks [75]). These are crucially impacted not only by carrier mobilities but also by the densities of mobile carriers. As far as the latter are concerned, it has been argued in the past that they can be massively impacted by the nature of the metal ions in the nodes [12,79]. Another factor massively changing free carrier concentrations in any type of semiconductor is the presence of extrinsic impurities (i.e., dopants) [12,27,80,81,82,83].

Besides chemical imperfections influencing the carrier concentration, structural imperfections can also have a tremendous impact—in this case, also on the carrier mobilities. The impact of some of these imperfections on the electronic coupling in Zn_2_(TTFTB) type systems is, therefore, discussed in the following section.

### 3.4. Role of Defects 

A consequence of the flat electronic bands along reciprocal space directions perpendicular to the TTF stacks (see Section 3.1) is that charge transport is essentially one-dimensional. It is well established for molecular semiconductors that transport in 1D systems is severely affected by either static or dynamic disorder [58]. This is not surprising, considering that an “obstacle” along a 1D transport path cannot be simply bypassed via neighboring sites. In the context of MOFs, it has, actually, been found that defects can lead to bands with almost no dispersion [49]. For the present systems, we considered several types of static defects. As mentioned above, dynamic disorder caused by vibrations of the MOF lattice is not considered here, although the defects discussed in the following, in some sense, also mimic what could happen as a function of the thermal motion of the MOF constituents.

**Figure 6:**
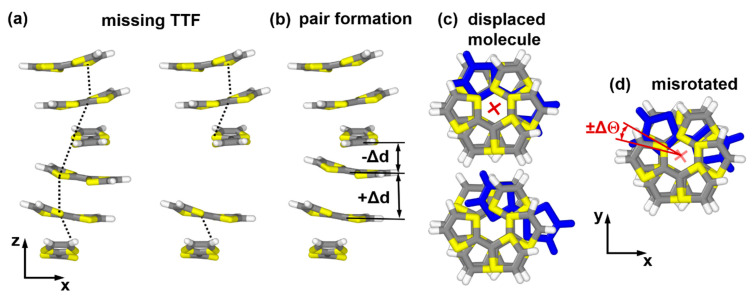
Instructive examples for possible structural defects in TTFTB-based MOFs. (**a**) Ideal model stack plus system with a missing TTF molecule; (**b**) structure of the system upon pair formation between neighboring TTFs; (**c**) displaced molecule defect realized by displacing one molecule along x; (**d**) structure of a misrotated molecule defect. It should be noted that for infinitely extended stacks, due to the employed periodic boundary conditions, a defect occurs in every unit cell.

**Figure 7:**
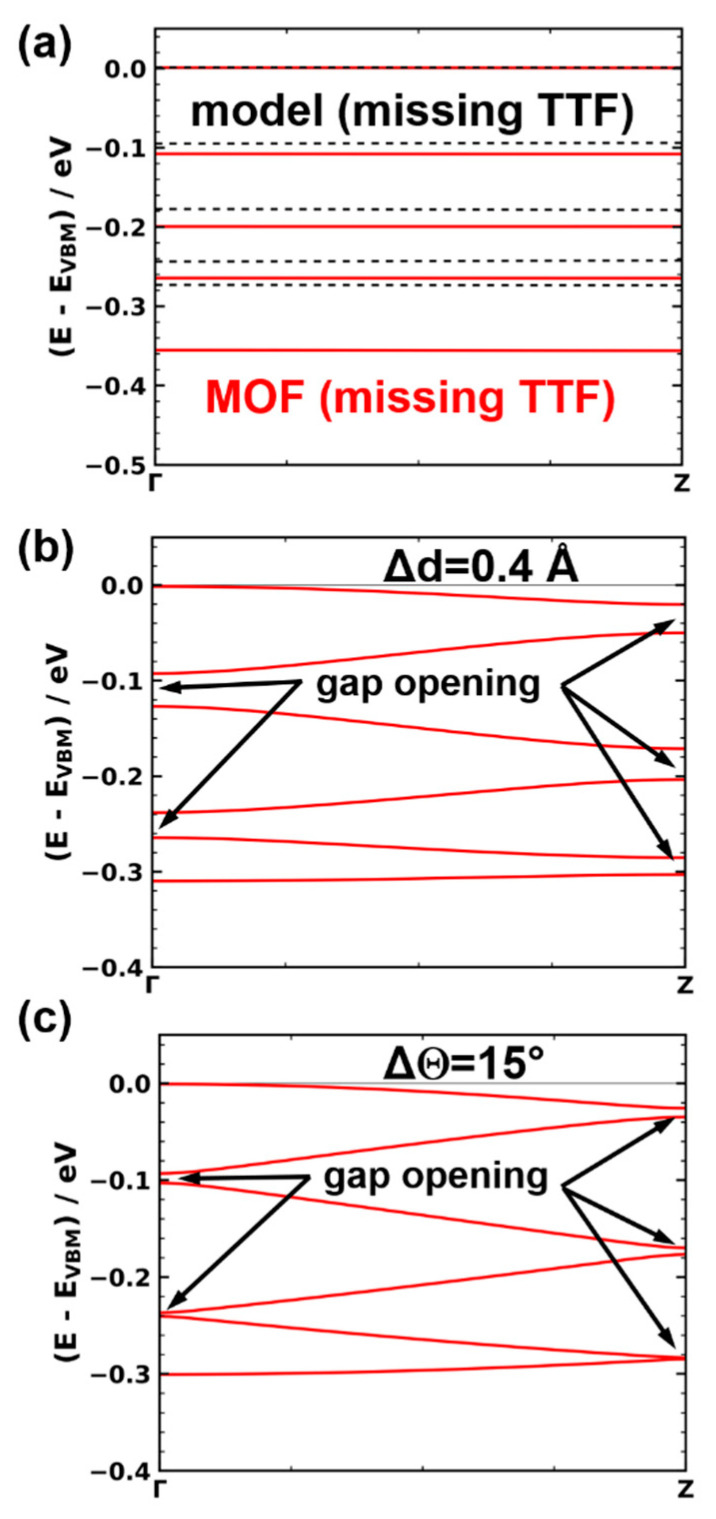
Electronic structure of the defective systems. (**a**) Electronic band structure in the stacking direction for Zn_2_(TTFTB) with a missing linker defect (red solid line). The results for the corresponding TTF stack are shown as dashed black lines. (**b**) Electronic band structure for the model TTF stack with a pair formation defect with a displacement of Δd = 0.4 Å. (**c**) Electronic band structure for the model TTF stack with a misrotated molecule defect of ΔΘ = 15°.

The static defect with the most dramatic consequences is a missing linker defect. We realized such a defect by removing one TTF linker from either the Zn_2_(TTFTB) unit cell or from the corresponding model stack (see Figure 6a). To describe pair formation as another type of defect, we displaced one molecule in the unit cell, such that it moved towards one of its neighbors by −Δd and away from the other neighbor by +Δd (see Figure 6b). A “displaced molecule” defect is characterized by one of the molecules in the unit cell being shifted from its equilibrium position along a vector parallel to the xy-plane (Figure 6c). In fact, it has been predicted for layered MOFs that interlayer displacements significantly affect the materials’ band structures [48,49,50]. For OSCs, it is also well known that changes in the intermolecular interactions induced upon variations of the involved molecule’s relative displacements depend on the actual shift direction [33,34,35,36,38]. In the present contribution, we focused on displacements along the x-direction (Figure 6b) as a representative example, highlighting the potential impact of such defects. For the final defect that is explicitly considered, the “misrotated” molecule case, the rotation angle of one of the molecules in the unit cell is changed by a value of ΔΘ (Figure 6d). Considering the structure of the MOF and identifying the degrees of freedom of each TTF moiety, one could actually identify several more structural defects. Examples are tilts of the molecules around the long and short molecular axes, changes in the bending of the molecules, or torsions around the central C=C bonds, to name a few. Therefore, a missing linker, pair formation, a “displaced molecule”, and misrotation of a molecule primarily serve as instructive examples for the possible impact of such structural defects on the electronic structure of the systems. Notably, the qualitative impact of all of the considered defects on the electronic structure of the model stacks is similar. They cause a loss of symmetry around the 6_5_ screw axis in the center of the stack. Consequently, the notion of a single TTF molecule as the “electronic” repeat unit of the stack no longer applies. In the band structure, this results in an opening of gaps at the Brillouin zone boundary and at the Γ point (see Figure 7 for the missing linker, pair formation, and misrotated molecule defects). Thus, for the defective structures, it is not sensible to report the width of the six-times backfolded valence band and we will instead focus on the effective masses at the valence band maximum. Additionally, in the spirit of hopping transport, we will report the smallest transfer integrals between neighboring molecules found in all inequivalent dimers extracted from each of the defective TTF stacks.

**Figure 8:**
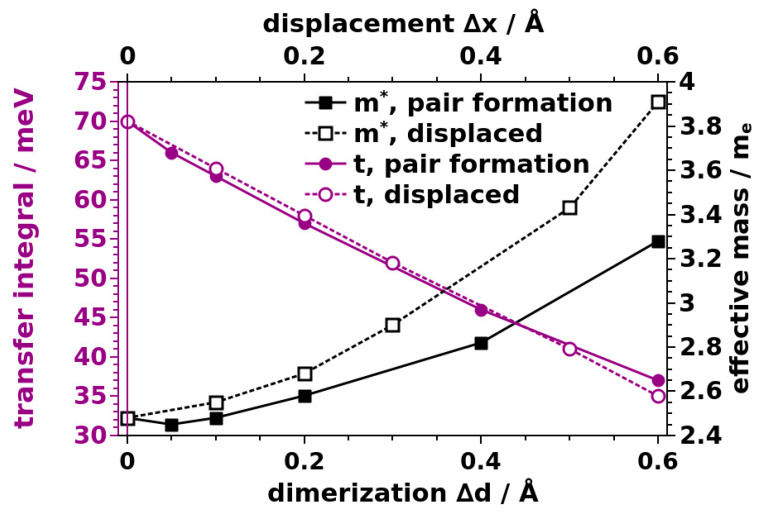
Evolution of the effective mass and the smallest transfer integral of the model TTF stack as a function of the dimerization displacement Δd and displacement Δx.

The missing linker defect has the most dramatic impact. It results in essentially flat bands (see Figure 7a), the minimum transfer integral drops to 1 meV, and the effective mass skyrockets to 22 m_e_. This shows that such a defect nearly stops charge transport along the affected TTF stack. As shown in Figure 8, also pair formation and displaced molecule defects result in an increased effective mass and a decreased transfer integral (with the exception of a minor decrease in m* for a very small dimerization of Δd = 0.05 Å, which is in the range of the uncertainty of the fitting procedure). The magnitude of the change increases with increasing displacement. Additional data on the impact of the other defects can be found in the Supplementary Materials. As a consequence, charge transport is hindered within defective TTF stacks. Interestingly, for pair formation as well as for the displaced molecule defect, the minimum transfer integral decreases almost linearly with the displacement, while the effective mass experiences a roughly quadratic increase. The latter is more pronounced in the displaced molecule case. The overall impact of these defects is, however, rather moderate (especially compared to the missing-linker case). For example, a lateral displacement of a TTF molecule by a rather sizable distance of Δx = 0.3 Å leads to an increase in m* by a moderate 0.42 m_e_ (or 17%).

In this context it, however, has to be considered that our test of defective structures is not exhaustive. Additionally, several defects might occur simultaneously, further worsening the situation. Nevertheless, the above considerations suggest that for changing the carrier mobilities by orders of magnitude, mere displacements of molecules might not be sufficient and more serious defects, such as missing linkers, are required. To the best of our knowledge, no systematic experimental study on the interplay between defect densities and (through-space) charge transport properties of MOFs has been carried out to date. However, especially for Zr-based MOFs (in particular for UiO-66) it is well known how to control the defect density, and extensive experimental and computational studies on the influence of the defect density on energy gaps, the redox conductivity, and the (photo-)catalytic activity have been carried out [84,85,86,87,88]. Therefore, such systems appear as prime candidates for also studying the impact of defects on charge-transport properties.

## 4. Conclusions

The present paper describes a variety of aspects concerning through-space charge transport in metal-organic frameworks in general and tetrathiafulvalene-based MOFs in particular. First, it is shown that the electronic band structure of the helical TTF stack contained in Zn_2_(TTFTB) largely determines the valence band structure of the entire MOF. In fact, we find that the electronic bands perpendicular to the TTF stacking direction are essentially flat. This highlights the negligible electronic coupling between neighboring stacks and establishes that Zn_2_(TTFTB) is a truly one-dimensional conductor. In the perfectly periodic MOF with six molecules in the crystallographic unit cell, the valence band is backfolded six times (without any gaps at the Γ-point or at the Brillouin zone boundary). This suggests that the symmetry element relevant for the electronic structure of the MOF is the six-fold screw axis parallel to the stacking direction. Therefore, a single TTF molecule acts as an “electronic” repeat unit of the MOF, with the consequence that the electronic parameter determining charge transport in Zn_2_(TTFTB) is the transfer integral between two neighboring TTF molecules.

This permits the use of stacks with varying numbers of molecules in the crystallographic unit cell to study the impact of the relative rotation of the TTF molecules. It turns out that decreasing the rotation angle of neighboring TTF molecules compared to the parent Zn_2_(TTFTB) system significantly increases the valence bandwidth, while increasing the rotation in a four-TTFs-per-unit-cell stack yields a significantly reduced electronic coupling. These results are corroborated by simulations on TTF dimers, which also allow us to trace the observations back to the shapes of the hybrid orbitals determining the valence band. Additionally, we found that the actual value of the transfer integral is extremely sensitive to the specific conformation of the TTF molecules. For example, for stacks of flat TTF molecules, the electronic coupling essentially disappears for the 60° rotation angles found in Zn_2_(TTFTB) and the associated transfer integral even changes sign at larger angles.

Interestingly, changes in the relative rotation and molecular conformation of the TTF molecules have a more pronounced impact on the observed bandwidth than “moderate” modifications in the stacking distance, which have been realized experimentally by replacing Zn with Cd atoms in the metal nodes of the MOFs. Thus, we hypothesize that the two-orders of magnitude increase in the electrical conductivity of Cd_2_(TTFTB) compared to Zn_2_(TTFTB) [53] must either be the consequence of significantly modified concentrations of mobile carriers or must be due to different defect densities in the two systems.

As far as static defects are concerned, we have, thus, investigated several scenarios, including displaced molecules, molecular pairing along the stack, or misrotations of specific molecules. The impact of these defects turned out to be rather moderate. This, however, changes when also considering missing linker defects, where we find that due to the 1D nature of the TTF stacks, such a missing linker is a massive obstacle for charge transport. This is manifested, e.g., in an increase in the effective mass by a factor of ~10 compared to the perfectly ordered parent MOF.

Overall, these results show that on the one hand, there is still considerable room for improvement for through-space charge transport in MOFs through clever structural design. On the other hand, the 1D nature of systems, such as the ones discussed here, makes their expected charge-transport properties particularly sensitive to structural imperfections and, thus, extremely dependent on sample quality.

## Figures and Tables

**Table 1:** Valence bandwidths (VBW) and effective masses at the valence band maxima for transport in (001) direction for all considered MOFs (i.e., the parent MOF Zn_2_(TTFTB), the MOF with Zn replaced by Cd, and the MOF with S replaced by Se) and for model TTF stacks with 1, 6, and 12 TTF molecules per unit cell (i.e., with rotation angles of 0°, 60°, and 30°). The systems TTF and TTFTB listed under MOFs are the stacks extracted from the Zn_2_(TTFTB) structure. For the model stacks, we compare systems generated with different geometries of the individual molecules. Here, (MOF) refers to TTF conformations extracted from the MOF structure, (relaxed) to geometries relaxed in the stack, (boat) to gas-phase relaxed TTF geometries in boat conformation, and (planar) to planar TTF molecules for which only the *x*- and *y*-coordinates have been relaxed in the gas phase.

	MOFs					
	VBW/meV			m*/m_e_		
Zn_2_(TTFTB)	371			2.05		
Zn_2_(TSFTB)	641			1.05		
Cd_2_(TTFTB)	333			2.21		
TTFTB	373			2.10		
TTF	303			2.40		
	**Model Stacks**					
	**VBW/meV**			m*/m_e_		
	**N = 1**	**N = 6**	**N = 12**	**N = 1**	**N = 6**	**N = 12**
TTF (MOF)	1337	298	650	0.93	2.48	1.84
TTF (relaxed)	1047	207	609	1.89	3.02	1.86
TTF (boat)	1269	72	348	1.23	7.29	4.35
TTF (planar)	1804	117	666	0.51	4.33	1.75

## Supplementary Materials

Additional data on the electronic structures of the considered systems as well as a description of the basis employed during the DFT calculations, details on the construction of the model systems, and validation of the simple tight-binding model are available online at https://www.mdpi.com/2079-4991/10/12/2372/s1. All calculations are available from the NOMAD database under https://dx.doi.org/10.17172/NOMAD/2020.11.25-1.
