# Supplementary material for: Strategies for Controlling Through-Space Charge Transport in Metal-Organic Frameworks via Structural Modifications
Source: Nanomaterials (Basel). 2020 Nov 28;10(12):2372. doi: 10.3390/nano10122372 (PMC7760313; doi:10.3390/nano10122372)
Supplement: Supplementary file 1 [file nanomaterials-10-02372-s001.pdf]

nanomaterials

Supplementary Material

Strategies for Controlling Through-Space Charge Transport in Metal-Organic Frameworks via Structural Modifications

Christian Winkler and Egbert Zojer *

Institute of Solid State Physics, NAWI Graz, Graz University of Technology, Petersgasse 16, 8010 Graz, Austria; christian.winkler@student.tugraz.at
* Correspondence: egbert.zojer@tugraz.at

1. Additional methodological details

1.1 Overview of basis functions used in FHI-AIMS

1.2 Construction of TTF and TTFTB model systems

1.3 Comparison of simple tight-binding model to electronic band structure of Zn_2(TTFTB)

2. Additional data for Zn_2(TTFTB) and the TTF model systems

2.1 Species projected and angular momentum resolved density of states of Zn_2(TTFTB)

2.2 Electronic structure of Zn_2(TTFTB) calculated with HSE06

2.3 Valence band of Zn_2(TTFTB) along additional high-symmetry k-space directions

2.4 Structure and Electronic Structure of Zn_2(TTFTB) with water

2.5 Conduction band of Zn_2(TTFTB) and model TTF stacks

2.6 Electronic structure of the TTFTB linker stacks

2.7 Electronic band structures of the considered TTF stacks

2.8 Electronic band structures deviating from simple tight-binding picture

2.9 Total energies of the optimized TTF stacks

2.10 Transfer Integral for a planar TTF dimer with a centered rotation axis

3. Data for additional systems considered: Cd_2(TTFTB), Zn_2(TTFTB)-Se

3.1 Electronic structure of Cd_2(TTFTB)

3.2 Electronic structure of Zn_2(TTFTB)-Se

4. Additional data for defects within the MOF and the model systems

4.1 Electronic structure of the defective TTF model systems

4.2 Electronic band structures for the dimerization defect data

4.3 Electronic band structures for the "displaced molecule" defect data

1. Additional methodological details

1.1. Overview of basis functions used in FHI-AIMS

Table 1. Basis functions that have been used for all calculations performed with FHI-AIMS. The abbreviations read as follows: H(nl,z), where H describes the type of the basis function where H stands for hydrogen-like type function, n is the main quantum number, l denotes the angular momentum quantum number, and z denotes an effective nuclear charge which scales the radial function in the defining Coulomb potential.[1].

	**H**	**C**	**S**	**O**	**Zn**	**Se**	**Cd**
Minimal	1s	[He]+2s2p	[Ne]+3s3p	[He]+2s2p	[Ar]+4s3p3d	[Ar]+4s3d4p	[Kr]+4d5s
Tier 1	H(2s,2.1)<br>H(2p,3.5)	H(2p,1.7)<br>H(3d,6)<br>H(2s,4.9)	ionic(3d,auto)<br>H(2p,1.8)<br>H(4f,7)<br>ionic(3s,auto)	H(2p,1.8)<br>H(3d,7.6)<br>H(3s,6.4)	H(2p,1.7)<br>H(3s,2.9)<br>H(4p,5.4)<br>H(4f,7.8)<br>H(3d,4.5)	H(3d,4.3)<br>H(2p,1.6)<br>H(4f,7.2)<br>ionic(4s,<br>auto)	H(2p,1.6)<br>H(4f,7)<br>H(3s,2.8)<br>H(3p,5.2)<br>H(5g,10)<br>H(3d,3.8)
Tier 2	H(1s,0.85)<br>H(2p,3.7)<br>H(2s,1.2)<br>H(3d,7)	H(3p,5.2)<br>H(3s,4.3)<br>H(3d,6.2)	H(4d,6.2)<br>H(4p,4.9)<br>H(1s,0.8)	H(3p,6.2)<br>H(3d,5.6)<br>H(1s,0.75)		H(4p,4.5)<br>H(4d6.2)<br>H(1s,0.5)	

1.2. Construction of TTF and TTFTB model systems

Additional details on the construction of several model systems discussed in the main manuscript are give below.

First, based on the relaxed structure of desolvated and dehydrated Zn_2(TTFTB), we constructed helical model TTF and TTFTB stacks by removing all atoms within the MOF structure apart from the TTF cores. The latter were then saturated by attaching H atoms. Subsequently, the positions of these H atoms were relaxed while keeping all other atomic positions fixed. This results in TTF molecules exhibiting the same geometry and stacking motif as in the MOF. These stacks were then arranged in the same pattern as in the MOF as shown in Fig. 1a of the main manuscript. In a similar way also the TTFTB model stack has been constructed: All atoms apart from the TTF core and the connected phenylene rings were removed. In essence, this means that the carboxyl groups have been removed from the linker in the MOF structure and replaced by H atoms. The positions of these H atoms then were relaxed in a subsequent step.

Second, for generating TTF stacks with different numbers of repeat units N, we simply picked one of the symmetry equivalent molecules of the parent stack discussed in the previous paragraph. This molecule was then duplicated, and rotated by the respective rotation angle ((n/N)*360°) around the off-center rotation axis (with n being an integer between 1 and N-1). Then the molecule was shifted in z-direction by n*3.473 Å. This procedure was repeated N-1 times to generate the unit cell for the simulations. For example, for 3 repeat units (N=3) we have in total three TTF molecules per unit cell including the molecule at the bottom of the cell (n=0) and its replicas at n=1 and n=2. The lateral extent of the unit cell of the model system is the same as that observed experimentally for Zn_2(TTFTB), while the extent of the unit cell in stacking direction amounts to N*3.473 Å. To verify the construction procedure we compared the electronic structure of the N=6 TTF model stack to the system extracted directly from the MOF structure. The identified differences are almost negligible (see Table 1) and can be assigned to subtle changes in the geometry of individual TTF molecules within the MOF-extracted system.

1.3. Comparison of a simple tight-binding model to the electronic band structure of Zn_2(TTFTB)

Here, we investigate whether a simple, one-dimensional tight-binding model with one "electronic" repeat unit can reproduce the 6-times backfolded band structure of the model stacks (shape and band width). This is relevant, as the transfer integrals reported in the main manuscript have been extracted from calculated actual band widths employing that approach. The model reads $E(k) = 2t * cos(k * R)$, with R being the shift between adjacent TTF molecules in the direction of the screw axis, i.e., the distance between electronic repeat units in stacking direction (R = 3.473 Å). From the electronic band structure of the 6 repeat unit TTF stack we extracted the transfer integral as (1/4)×W, with W being the calculated band width. Using W=298 meV we then calculated the electronic band structure of the model. In order to compare it to the 6 times backfolded band structure of the TTF stack we folded the resulting band back into the crystallographic unit cell of the TTF stack containing 6 molecules. Comparing the two band structures (green for model and red for TTF stack in Figure S1a), we find an excellent qualitative agreement. I.e., the simple tight-binding model with a single TTF molecule as "electronic repeat units" provides a physically meaningful model for the valence band of the TTF stack. Also the quantitative agreement is reasonably good, which supports the use of t = W/4 for depicting the trends for the transfer integrals in the main manuscript.

In the actual stack, there might, of course, also be couplings to next nearest neighbors which might play a role. Such couplings could be hyperexchange-like, as we observed it in quinacridone, a prototypical H-bonded organic semiconductor.[2] Therefore, we also considered a slightly modified model, where we considered next-nearest neighbor couplings as well. It reads: $E(k) = 2t * cos(k * R) + b * 2t * cos(2 * k * R)$. The coupling between these sites was estimated to be around 10% of the nearest neighbor couplings (~8 meV) where b actually is a fit parameter (smallest RMSE for b=0.1). A comparison between the bands resulting from this model and the actual TTF stack is shown in Figure S1b. There, we observe a further improved agreement between the simple tight-binding model and the data of the actual TTF stack, especially at the band extrema.

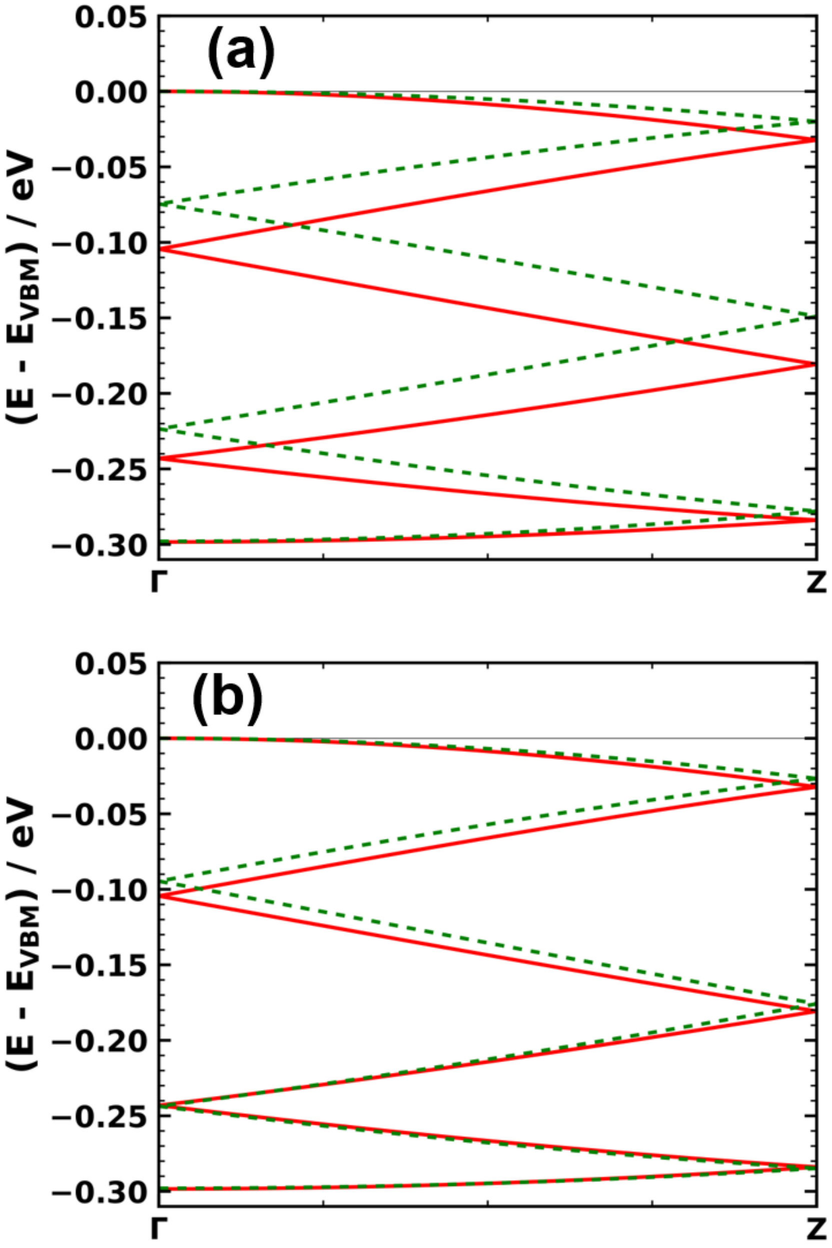

Figure S1. Comparison of band structures resulting from simple tight-binding models (green dashed line) and the 6 repeat unit TTF stack (solid red line). (a) One-dimensional tight-binding model with only nearest neighbor couplings considered. (b) One-dimensional tight-binding model with next-nearest neighbor couplings also included.

2. Additional data for Zn_2(TTFTB) and the TTF model systems

2.1. Species projected and angular momentum resolved density of states of Zn_2(TTFTB)

For a further in-depth analysis, we projected the density of states onto individual parts of the MOF: the central TTF stack (TTF), the phenylene rings (rings) and the metal nodes (frame). Similar to the findings in Ref [3], this projected density of states (pDOS, displayed in bottom-right panel of Figure S2) shows that the valence band is primarily formed from states localized on the TTF stacks. Analyzing the angular momentum resolved species projected DOS also shown in Figure S2 we find that these states are of p-character. The phenylene rings contribute only weakly to the valence band. This is consistent with the notion that the valence band describes a through-space pathway for holes along the helical stack of TTF molecules. In contrast, for the conduction band, one can see non-negligible contributions from the phenylene rings as well as from the metal nodes. This means that electron transport also involves other parts of the MOF besides the central TTF stack. For the conduction band also O p-states and a small contribution from Zn p-states are important.

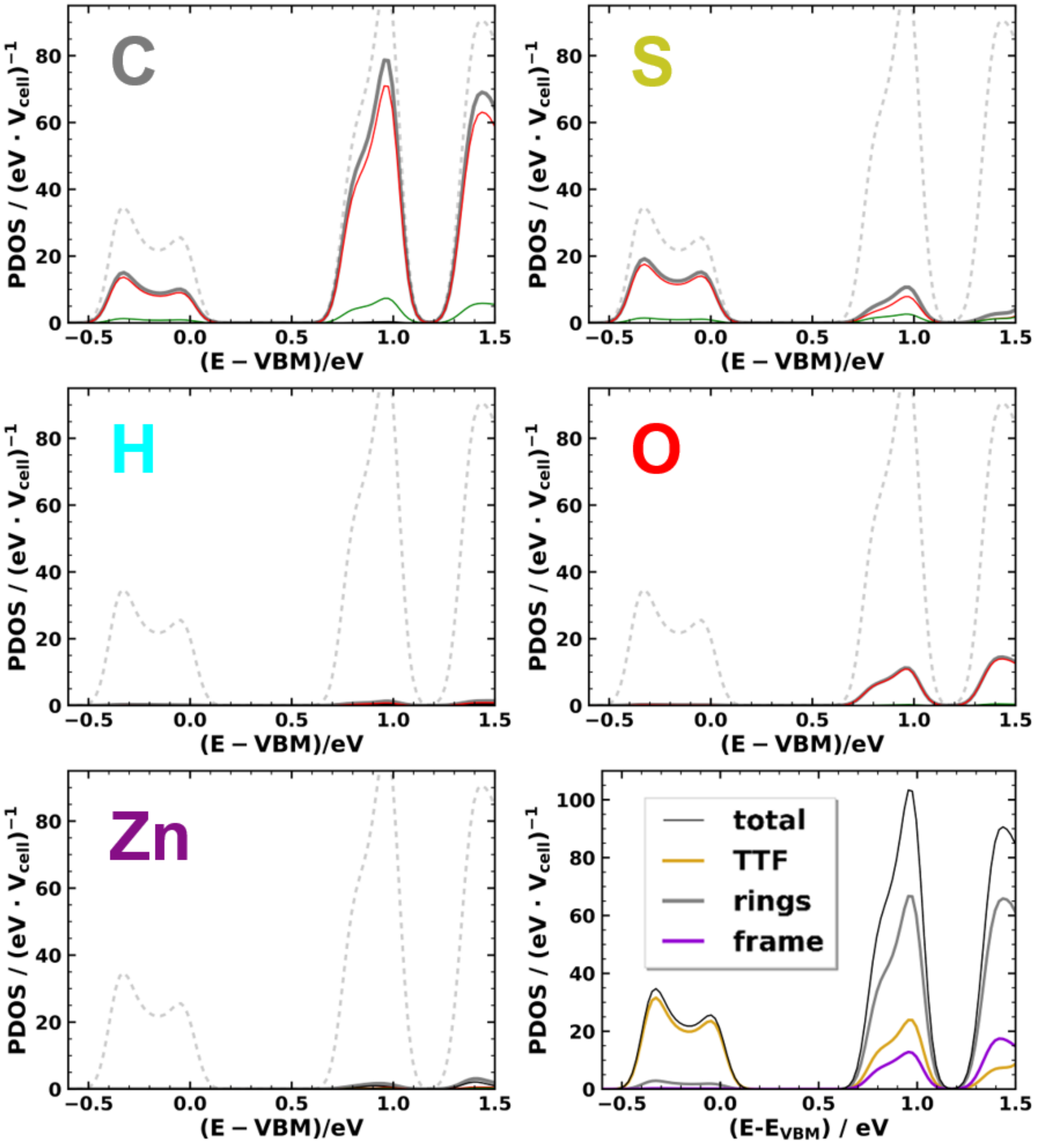

Figure S2. Species-projected and angular momentum resolved density of states for Zn_2(TTFTB). Grey is the total contribution of the individual species, black indicates s-states, red p-states and green d-states. Bottom right panel: Density of states of Zn_2(TTFTB) projected onto individual parts of the

MOF. “TTF” refers to the TTF units, “rings” to the attached phenylene rings and “frame” to the nodes. The total density of states (as the sum of the individual contributions) is given by the black line.

2.2. Electronic structure of Zn_2(TTFTB) calculated with HSE06

To see how the electronic structure of the MOF would be affected by the choice of the actual functional (in particular the treatment of exchange and correlation), we also employed the range separated hybrid functional HSE06 to calculate the electronic structure of Zn_2(TTFTB) (Figure S3). As expected for hybrid functionals, this results in an increased band gap (0.773 eV for PBE and 1.483 eV for HSE), which is, however, of no relevance for the discussion in the present manuscript. More relevant is the slightly larger width of the valence band obtained with HSE, but the overall effect is rather minor. These two differences aside, there is virtually no difference between the PBE and the HSE calculations.

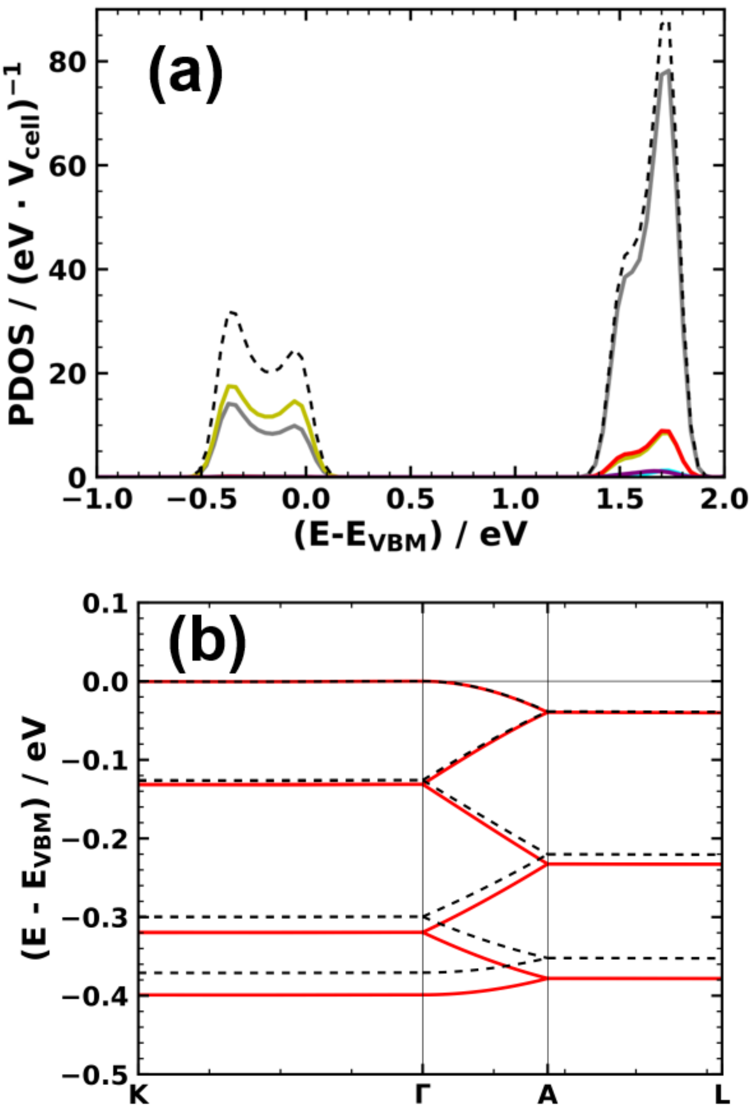

Figure S3. Electronic structure of Zn_2(TTFTB) calculated employing the HSE06 functional and using a PBE-optimized geometry. (a) Species projected density of states (C—grey, H—cyan, O—red, Se—yellow, Zn—purple) and (b) Electronic band structure of the valence band as obtained with HSE06 (red) compared to the PBE result (black, dashed).

2.3. Valence band of Zn_2(TTFTB) along additional high-symmetry k-space directions

To investigate whether the valence band is flat for all directions apart from those associated with the stacking direction of the TTF cores (GA and LM), we calculated the electronic band structure along a path considering all relevant high-symmetry k-space directions (see Figure S4). Indeed we find that only for directions parallel to the stacking direction of TTF the valence band shows a significant dispersion.

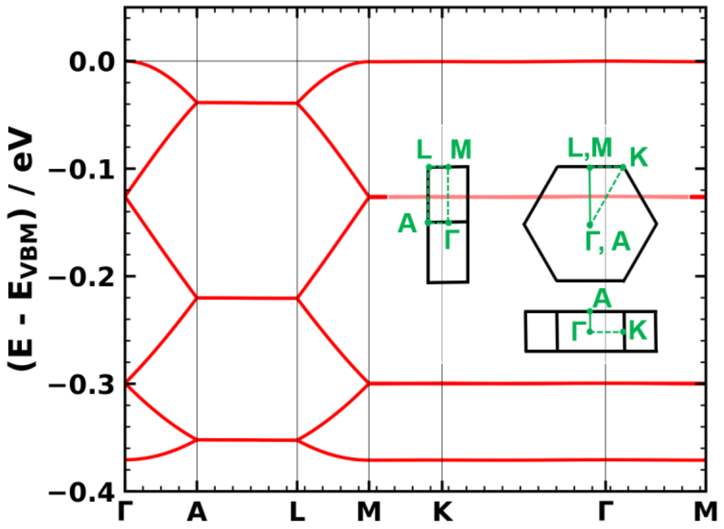

Figure S4. Electronic band structure for Zn_2(TTFTB) for a path covering all relevant high-symmetry directions in the first Brillouin zone. The first Brillouin zone is shown as an inset.

2.4. Geometrical and electronic structure of Zn_2(TTFTB) containing water

To investigate the influence of water molecules coordinated to the Zn atoms in the Zn_2(TTFTB) MOF, we considered the initial structure extracted from the cif-file from reference [3], removed the solvent molecules and calculated the electronic structure of this system. The used geometry and the obtained electronic structure are shown in the left panel of Figure S5. In the top panel, the structure with the water molecules extracted from the cif file is compared to the structure that has been obtained by relaxing the atomic positions of Zn_2(TTFTB) without water (green). One can see that there are only slight differences in the geometric structure of the systems. Considering the electronic band structure of the valence band for this geometry we find a valence band width of 353 meV and a corresponding effective mass of 1.97 m_e. Both compare well to the 371 meV and 2.05 m_e obtained for the optimized Zn_2(TTFTB) structure without water.

The results for the fully relaxed atomic positions calculated including the water molecules are compared to the relaxed geometry of the system without water (right panel in Figure S5 and Figure S6 a). We find that the central TTF core is hardly affected by the presence of water molecules at the Zn coordination sites, while the phenyl rings indeed show structural changes. As outlined in the main manuscript also these phenyl rings contribute to the valence band. Thus, considering the electronic structure of the relaxed MOF with water we find that this system exhibits a larger band width of 411 meV (1.76 m_e) compared to the system without water (see Figure S6 a for band structures). Nevertheless, these changes are quite minor. Therefore, the system without water can serve as a prototypical example for the influence of structural effects on the charge transport properties. Additionally we tested, whether one can use a TTF model system (constructed according to section 3.2 in the main paper) for describing the valence band of the relaxed system with water (dashed black line in Figure S5). Indeed we find that such a TTF stack can serve as a viable model system, only slightly underestimating the resulting valence band width of the actual MOF. For the sake of completeness we also report the species projected DOS of the relaxed Zn_2(TTFTB) MOF with water molecules coordinating to Zn in Figure S6.

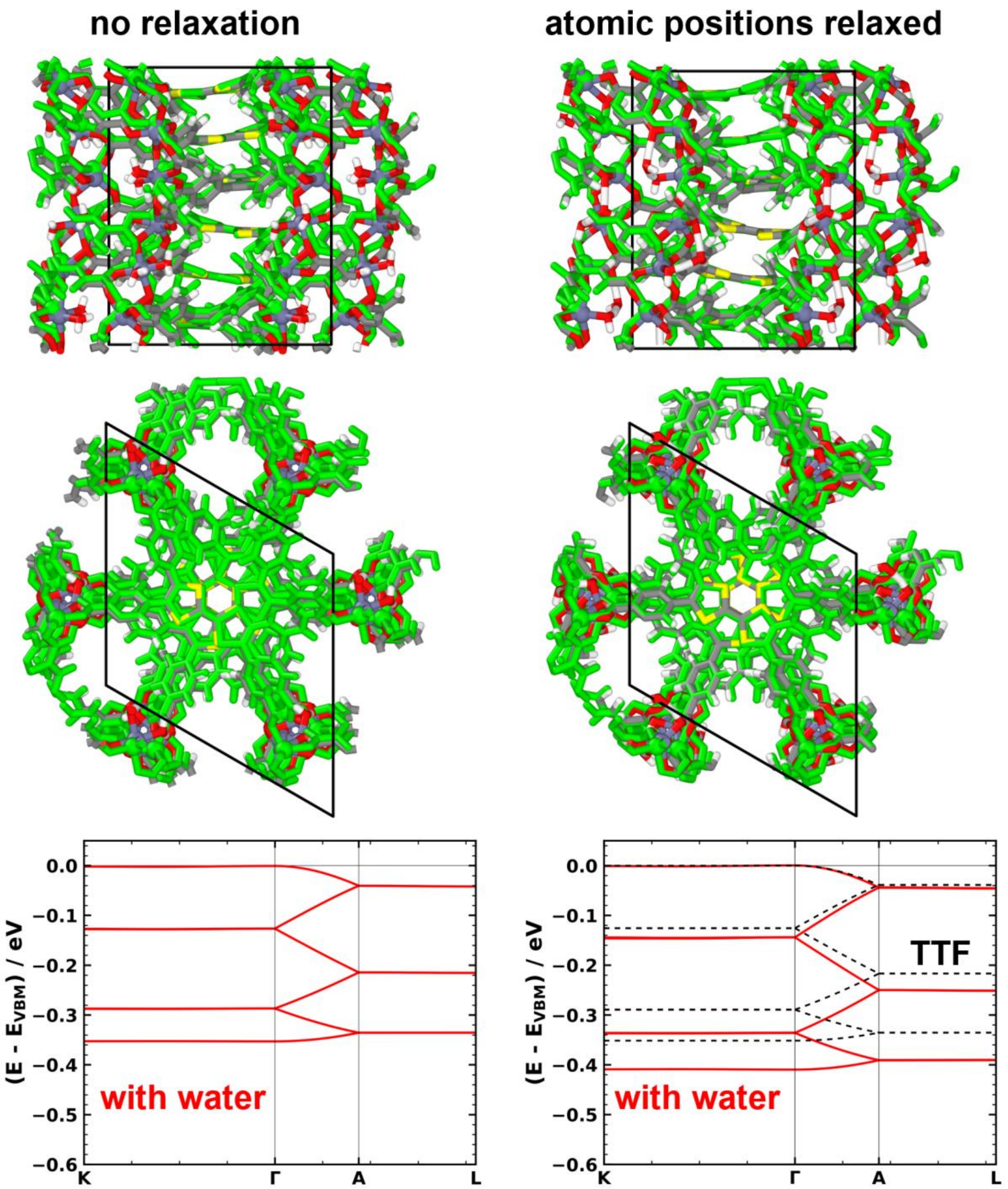

Figure S5. Geometric structure and electronic band structure of Zn_2(TTFTB) with and without water molecules coordinated to the Zn metal atoms. Two systems are considered and compared to the structure obtained by relaxing the atomic positions without water molecules present (green): The left panels show data for a structure with water, as reported in literature cif file (i.e., without a further geometry relaxation). The electronic band structure is shown below the crystallographic structure. The right panel shows the structure with water after performing a geometry relaxation of the atomic positions. In the bottom panel the electronic band structure of that system is shown in red. The dashed black line corresponds to the data for the saturated TTF stack including water molecules. The unit cell is shown by the solid black lines in the geometric structures.

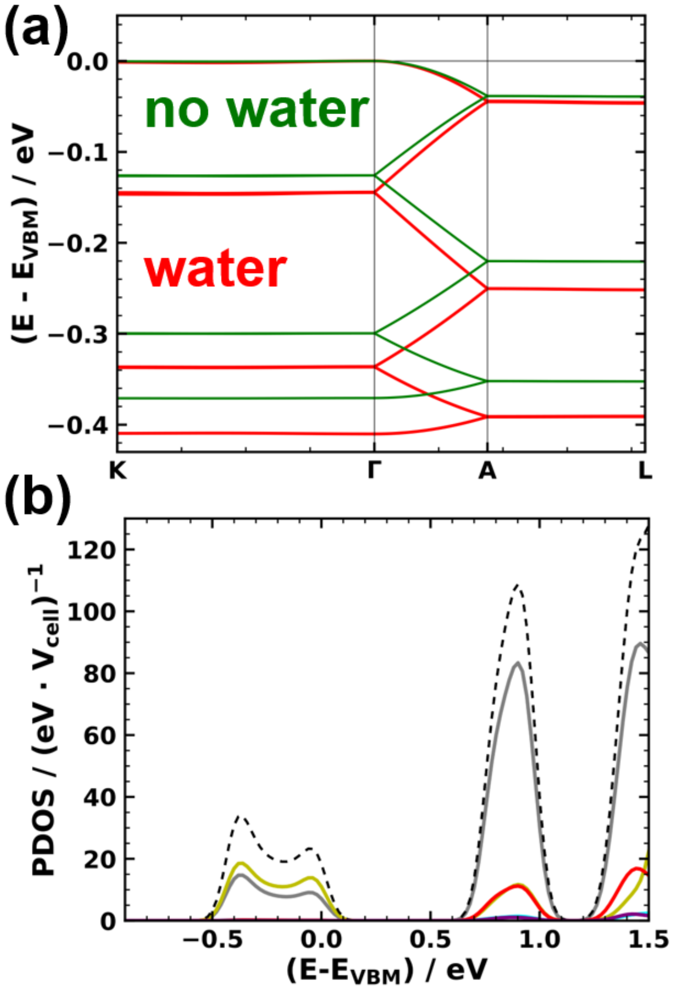

Figure S6. Electronic structure of Zn2(TTFTB) of relaxed Zn_2(TTFTB) with water molecules coordinated to Zn. (a) Electronic band structure of Zn_2(TTFTB) with water (red) compared to Zn_2(TTFTB) without water (green). (b) Species projected density of states of Zn_2(TTFTB) (C—grey, H—cyan, O—red, Se—yellow, Zn—purple, total—dashed).

2.5. Conduction band of Zn_2(TTFTB) and model TTF stacks

Considering the conduction band of Zn_2(TTFTB) (without water; mFigure S7) we find that it has a significantly smaller band width than the valence band. This has already been discussed in the main manuscript. Additionally, we can observe that bands along directions perpendicular to the stacking direction (KΓ and AL) exhibit small dispersions of around 20 meV. This means that unlike for the valence band, for the conduction there is a small coupling between neighboring TTF stacks. This coupling is potentially mediated by Zn and O p-states, as can be rationalized by these atoms'/orbitals' contributions to the conduction band.

Considering that not solely states arising from the central TTF core contribute to the conduction band it is not surprising that a model stack consisting only of TTF molecules cannot reproduce the conduction bands of the actual MOF (see dashed line in Figure S7).

Interestingly, for the system with water (after relaxing the atomic positions), we find that the dispersion along KΓ and AL is significantly reduced (see Figure S8). This indicates a weaker coupling between neighboring TTF stacks for the conduction band in the presence of water.

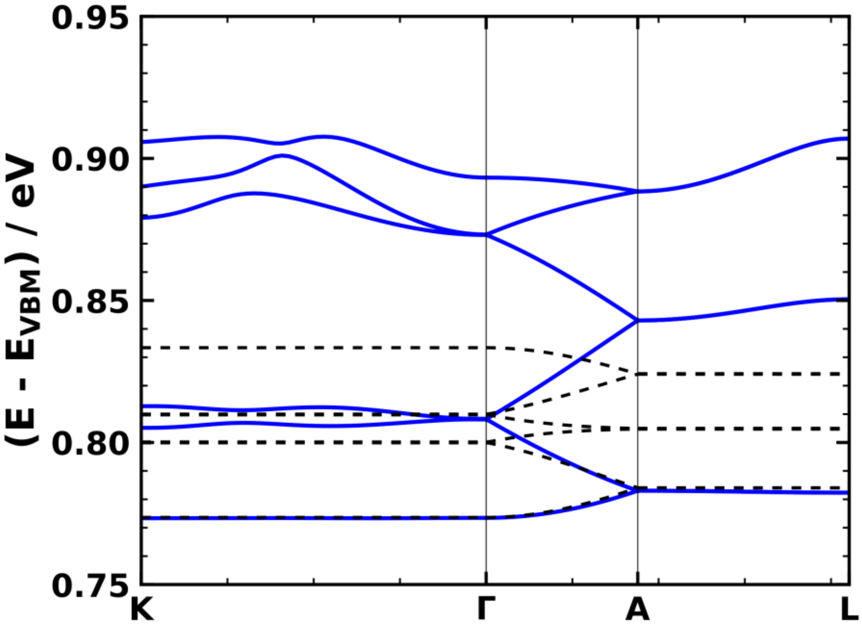

Figure S7. Electronic band structure of the conduction band for Zn_2(TTFTB) in blue and the corresponding TTF model stack as the black dashed line. One can see that bands along reciprocal space directions perpendicular to the TTF stacks show a small dispersion (~20 meV), which means that for the conduction band there is a small coupling between neighboring stacks.

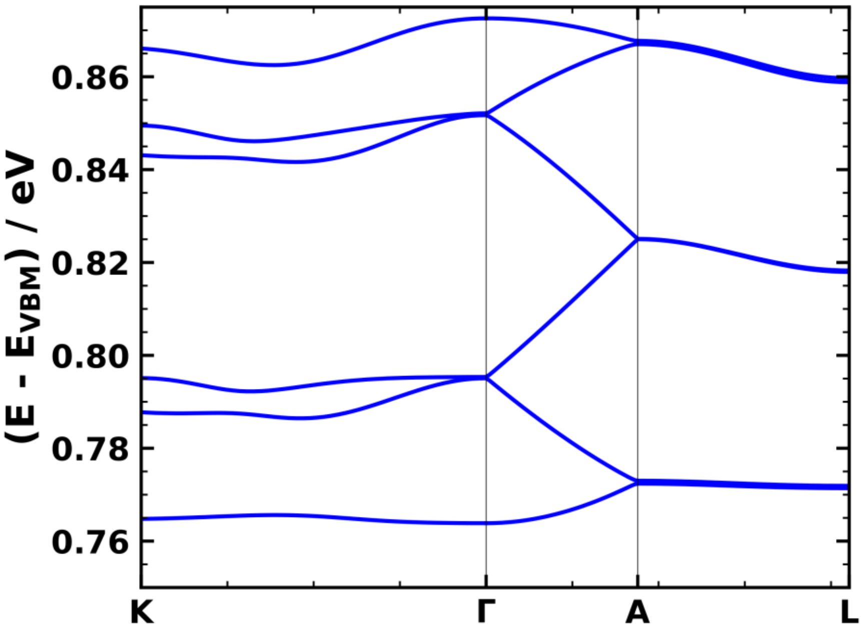

Figure S8. Electronic band structure of the conduction band for Zn_2(TTFTB) with water coordinated to the Zn atoms. One can observe that in comparison to the system without water, the dispersion along directions perependicular to the TTF stacks is reduced significantly.

2.6. Electronic structure of a stack consisting of the central TTF core of the TTFTB linkers plus the phenylene rings

From the projected density of states in the main manuscript one can already conclude that the phenyl rings show a non-negligible contribution to the valence band of Zn_2(TTFTB). To show this in a more explicit way we, extracted a stack of TTFTB molecules from the MOF and replaced the carboxyl groups with H (see structure in Figure S9). After relaxing the atomic positions of these H atoms we calculated the electronic band structure of this model system. Comparing the valence bands of the model system and of the full Zn_2(TTFTB) MOF in Figure S9 reveals an excellent agreement between the band structures of the two systems.

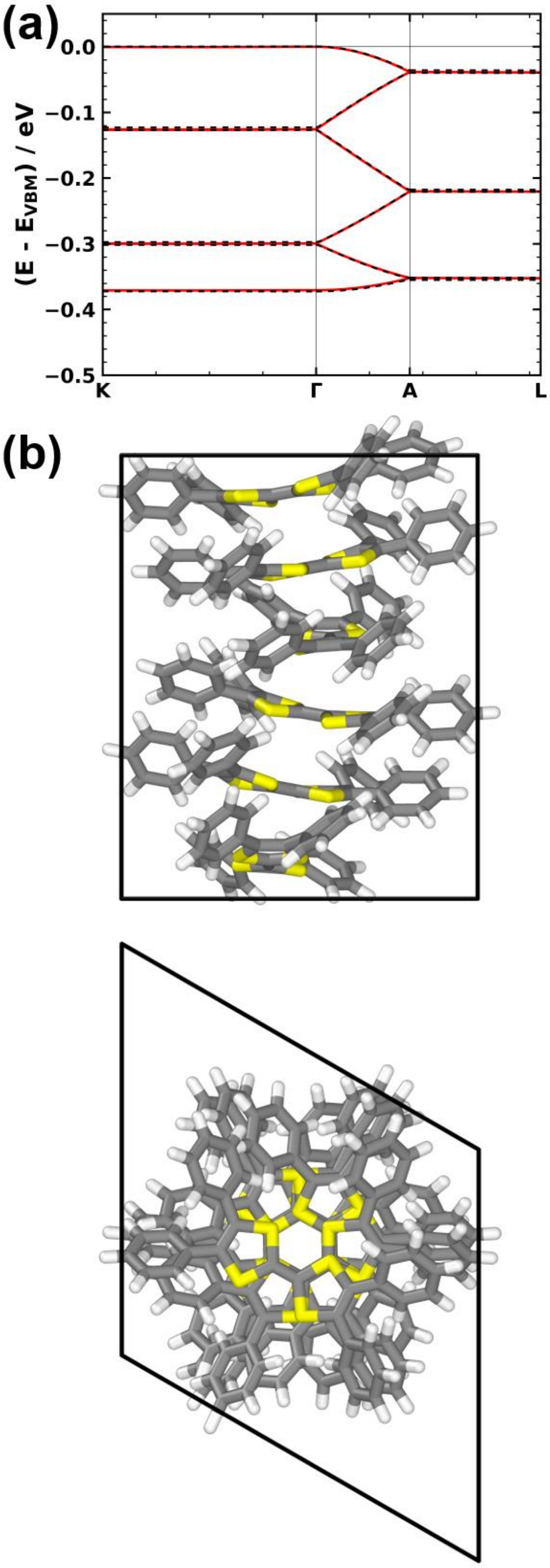

Figure S9. (a) Electronic band structure of Zn_2(TTFTB) in red and of the isolated and saturated TTFTB stack (black). (b) In the lower panel the unit cell of the TTFTB stack is shown.

2.7. Electronic band structures of the considered TTF stacks

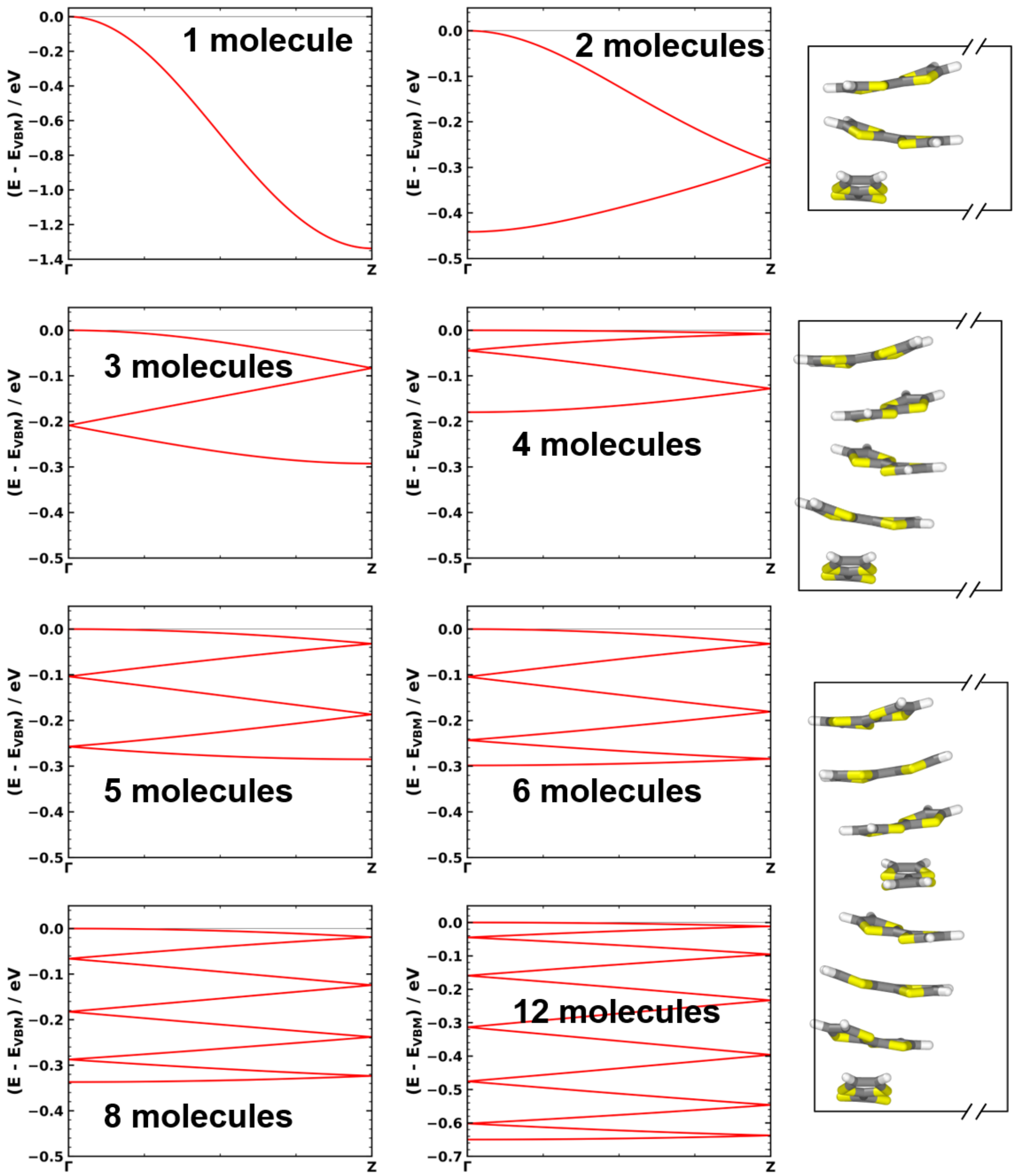

Figure S10. Electronic band structures of the TTF model stacks with 1, 2, 3, 4, 5, 6, 8, 12 repeat units. In the right panel unit cells for systems with 3, 5, and 8 repeat units are shown as examples.

2.8. Electronic band structures deviating from the simple tight-binding picture

For certain systems we found that the electronic band structure of the valence band deviates from the shape one would expect for a simple 1D tight-binding model (see section 1.2 of SM). For relaxed with 4, boat and planar molecular geometries with 5 repeat units we find that the band maximum is slightly off Γ, but with an energy difference significantly smaller than 25 meV (k_BT at room temperature). In these systems the n-fold backfolded valence band splits into two electronic bands. For determining the band width we, thus, consider the entire energy range covered by these

bands. For planar TTF with 3 repeat units we see that the band has picked up a contribution with a higher frequency, meaning that electronic couplings beyond the nearest neighbor become relevant.[2]

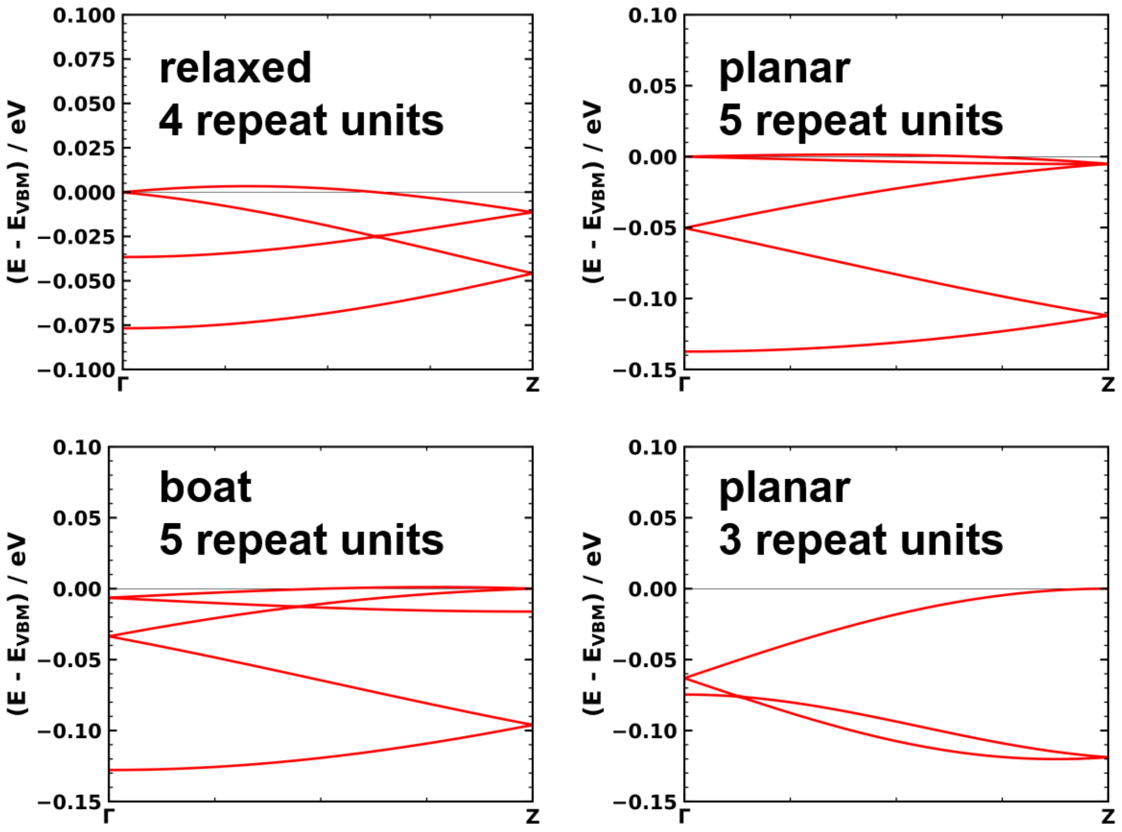

Figure S11. Electronic band structure of the stacks marked by black frames in Figure 3 of the main manuscript. For these systems we find that the electronic band structures deviate from the shape one would expect from a simple 1D tight-binding model with a single TTF molecule as "electronic" repeat unit.

2.9. Total energies of the optimized TTF stacks

Considering the total energies per TTF repeat unit of the relaxed TTF model stacks (see Figure S12a) we find that except for the cofacial system (1 repeat unit) and the the 2 repeat unit system all stacks exhibit energies within less than 35 meV. Considering the planar TTF model system shown in Figure S12b one can find a correlation between the total energy and the transfer integral (band width), especially for large rotation angles. Such a behavior is, in fact, not unexpected considering the role of exchange interactions as described in [4]. One can, however, also observe that the vdW interactions between the TTF molecules play an important role in determining the energetic stability of certain arrangements (TTF stacks). For the fully optimized structures, such a correlation is less pronounced, which is a consequence of different distortions of the molecules at different rotation angles significantly changing the distances between atoms in neighboring molecules.

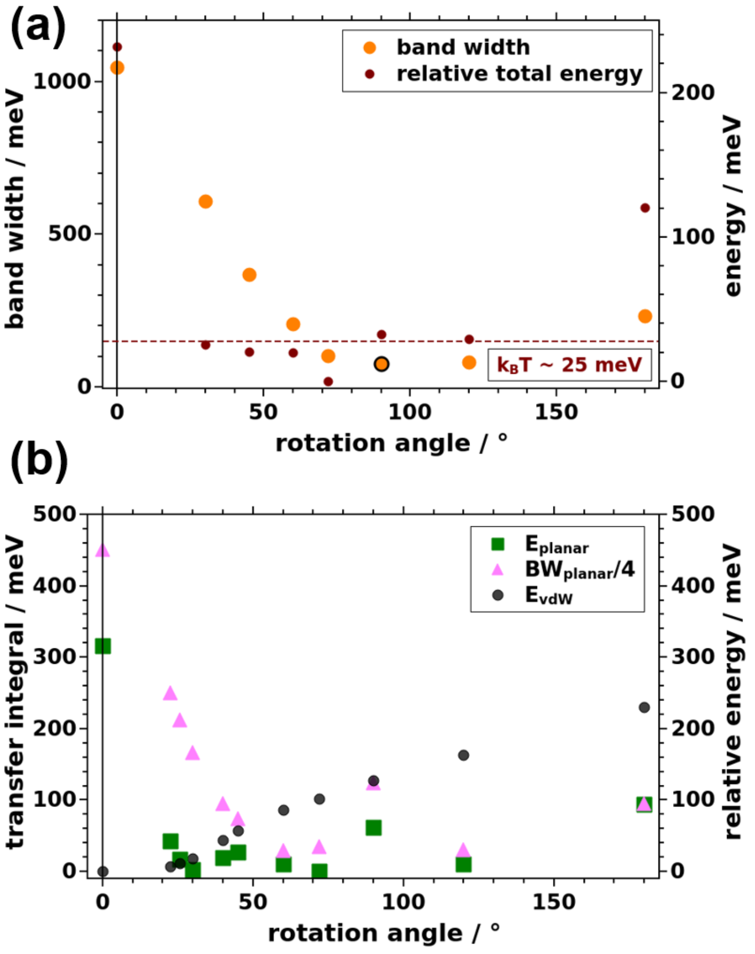

Figure S12. Evolution of the band width (transfer integral) and the total energy per TTF molecule for theTTF model stacks. (a) Band width and relative total energy for the relaxed TTF model stack. (b) Transfer integral as (1/4)* valence band width, relative total energy, and vdW energy for the planar TTF model stack. For the planar model stack additional data points for 9, 14, and 16 repeat units were added. This was done to clarify the evolution of the relative total energy for angles between 20° and 50°. The energies are aligned to the global minimum, this means that positive energies result in less stable arrangements.

2.10. Transfer integral for a planar TTF dimer with a centered rotation axis

To investigate the influence of the position of the rotation axis on the evolution of the transfer integrals we considered a planar TTF dimer with the screw axis connecting the centers of the planar molecules. Varying the relative rotation angles of the two monomers with respect to this rotation axis results in the evolution shown in Figure S13a. The data points for a rotation angle of 0° are the same as for the planar geometry in Figure 3b of the main manuscript and one again observes a decrease of the coupling with increasing rotation angle. The absolute magnitude of the coupling is minimized for an intermediate rotation (~50°). In contrast to the situation for the off-center screw axis in Figure 3b, for a centered screw axis one never observes a change in the signs of the band widths and transfer integrals. This can be traced back to the fact that the orbitals never change their order, consequently also the transfer integral always exhibits the same sign. The respective orbitals for the extrema of the transfer integral are shown in Figure S13b.

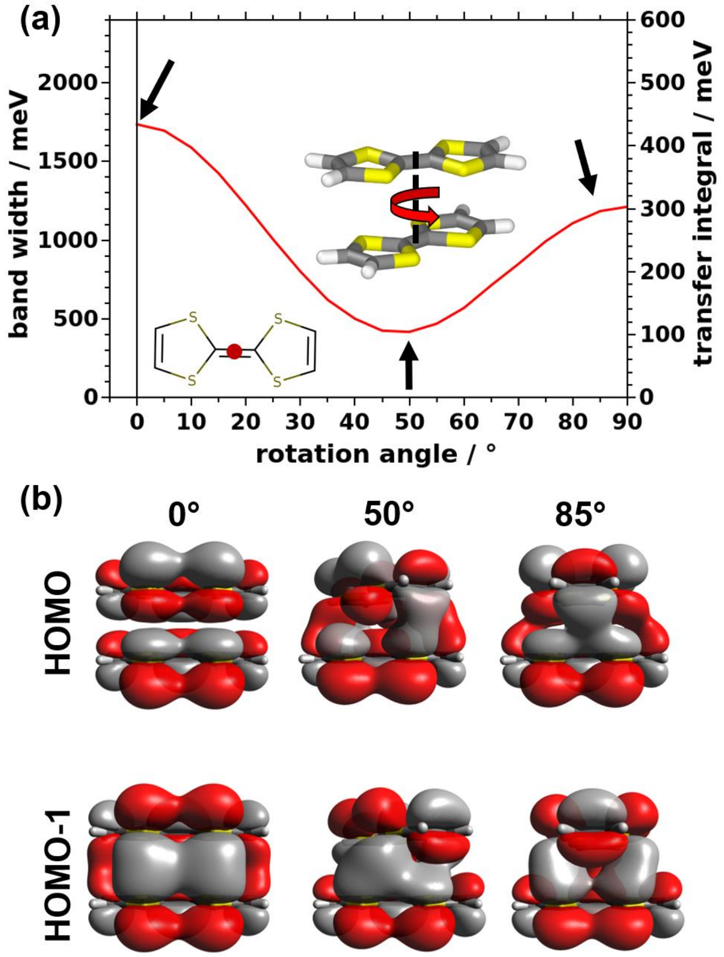

Figure S13. Electronic structure of Planar planar TTF dimers with the rotation axis placed in the center of the TTF molecules. Panel (a) shows the evolution of the band-width and the transfer integral as a function of rotation angle. Panel (b) shows the molecular orbitals (HOMO and HOMO-1) for rotation angles corresponding to local the extrema of the band-width (marked with black arrows).

3. Data for additional systems considered in the current manuscript: Cd_2(TTFTB), Zn_2(TSFTB)

3.1. Electronic structure of Cd_2(TTFTB)

As an additional MOF system considered here is Cd_2(TTFTB) (structure from ref [3]), which is isostructural to Zn_2(TTFTB). For this system we relaxed the atomic positions within the reported unit cell[3] and then calculated the electronic band structure for this relaxed geometry. The resulting species projected density of states and the valence band structure are shown in Figure S13.

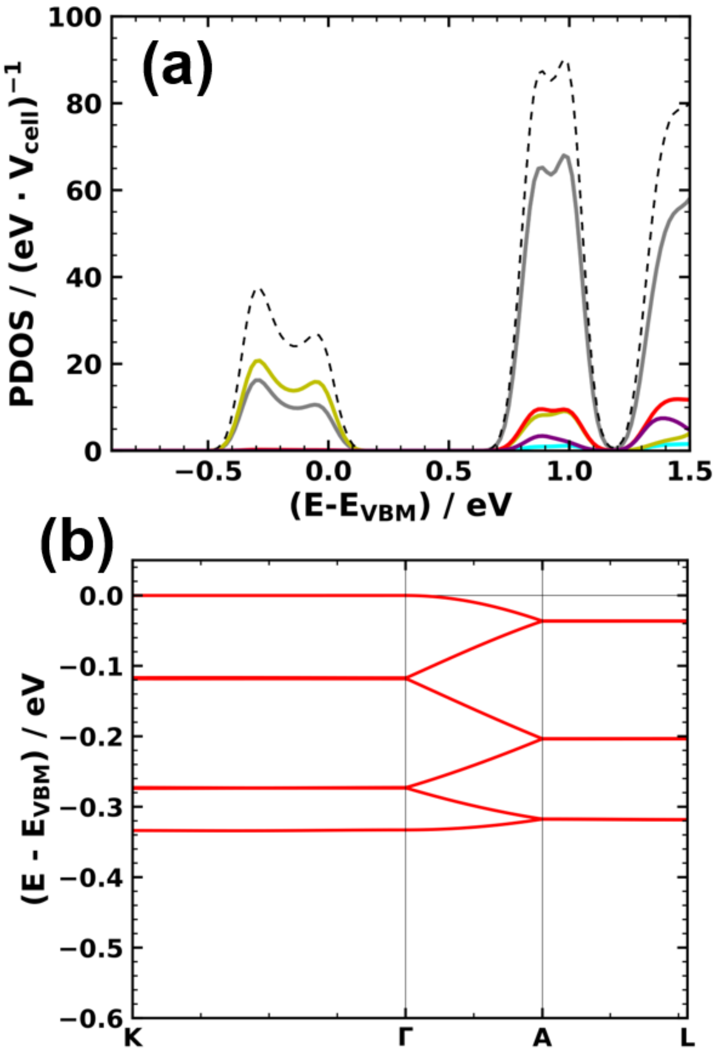

Figure S14. Electronic structure of the Cd_2(TTFTB) MOF. (a) species projected density of states (C—grey, H—cyan, O—red, Se—yellow, Cd—purple) and (b) valence band aligned to its maximum.

3.2. Electronic structure of Zn_2(TSFTB)

Also the electronic structure (PDOS and electronic band structure) of a MOF with Tetraselenafulvalene ($C_6H_4Se_4$) replacing TTF has been calculated. The structure of this system was obtained by taking the structure of Zn_2(TTFTB) and replacing S with Se, i.e. replacing TTF with TSF. As no experimental cell for this system exists and as we expect the larger p-orbitals of Se to cause an increase of the stacking distance, we relaxed the atomic positions of the starting geometry as well as the cell vectors. For comparison we also calculated the electronic for the system when relaxing the atomic positions while keeping the unit cell fixed. The obtained species projected density of states and the structure of the valence band for the system with the relaxed unit cell are shown in Figure S15. The data for the system with relaxed atomic positions but within the unit cell of Zn_2(TTFTB) are

shown in Figure S16. For the fully relaxed system we obtain 678 meV for the valence band width and an effective mass of 0.98 m_e. For the system in the Zn_2(TTFTB) unit cell we get 641 meV and 1.06 m_e, so there is hardly any difference between the relevant quantities of these systems.

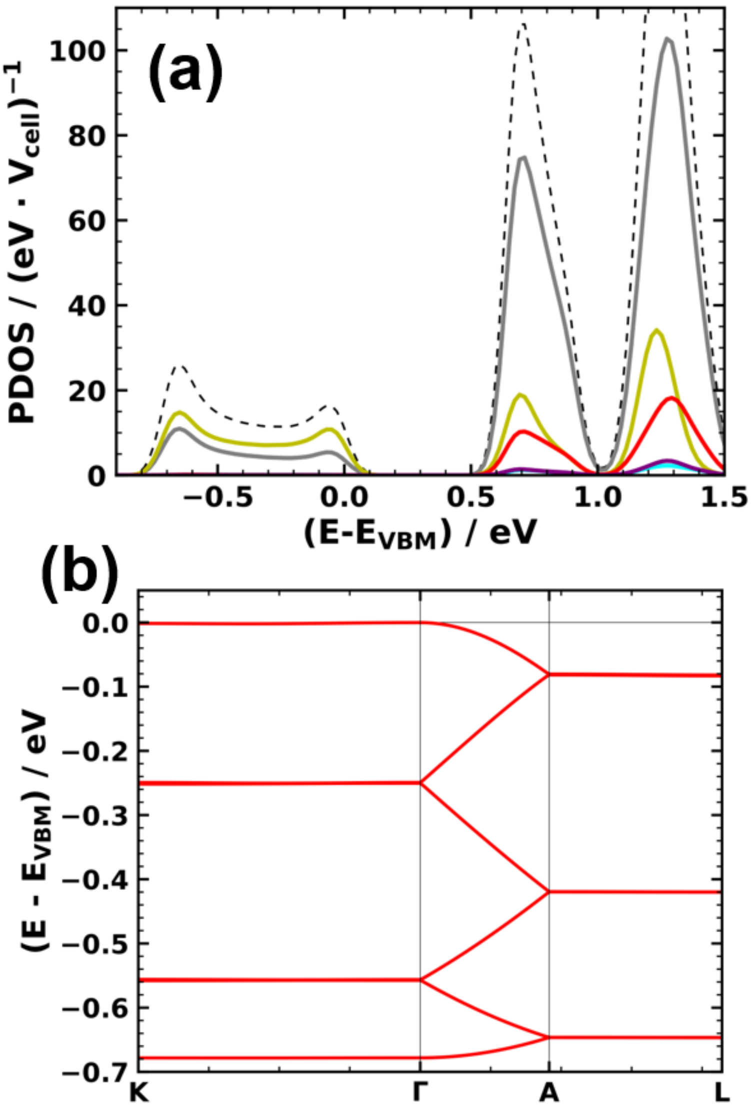

Figure S15. Electronic structure of the Zn_2(TSFTB) MOF for the relaxed unit cell. (a) Projected density of states (C—grey, H—cyan, O—red, Se—yellow, Zn—purple, total—black dashed) and (b) electronic band structure of the valence band aligned to its maximum.

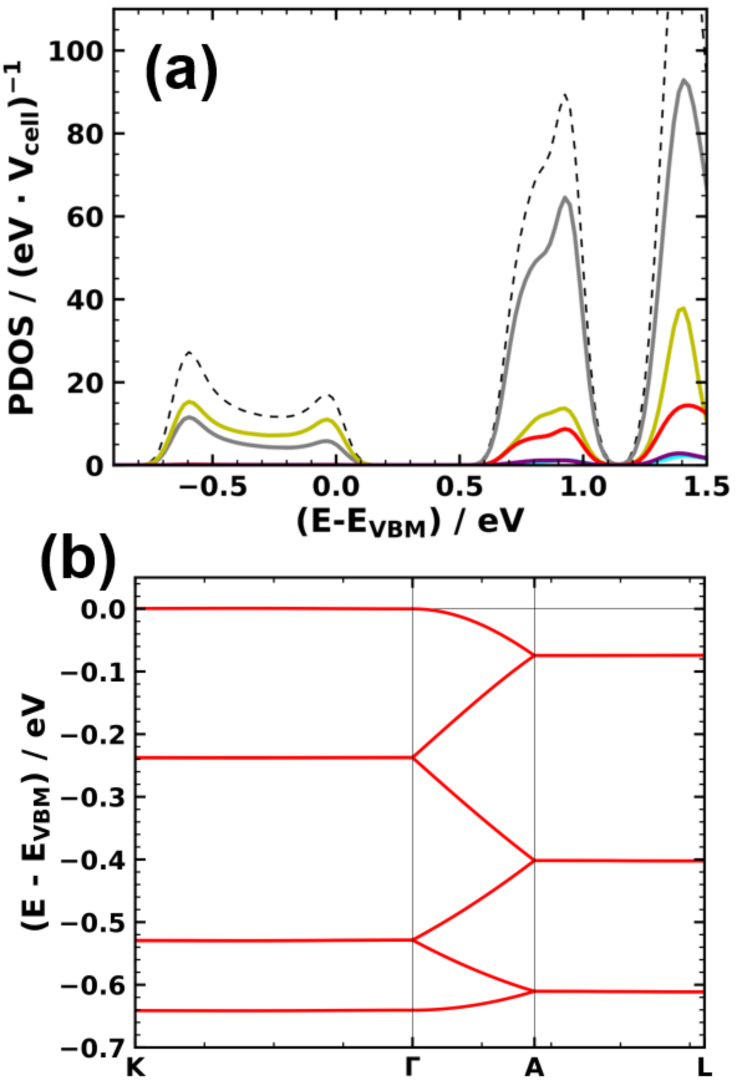

Figure S16. Electronic structure of Zn_2(TSFTB) MOF in the unit cell of Zn_2(TTFTB). (a) Projected density of states (C—grey, H—cyan, O—red, Se—yellow, Zn—purple, total—black dashed) and (b) electronic band structure of the valence band aligned to its maximum.

4. Additional data for defects within the MOF and the model systems

4.1. Electronic structure of a TTF model systems with a displaced rotation axis

To show the influence of other potential defects we considered also shifts of the center of rotation for one of the TTF molecules. The resulting band structures are shown in Figure S17. Again one can see that upon introducing of such defects a gaps open at the BZ boundary and at the G point. These gaps and the resulting changes of the band dispersion y lead to an increase of the effective mass (Table S2), i.e a decrease of the transfer integral – similar to the data presented in the main manuscript.

Table 2. Effective mass depending on the offset of the rotation center of one of the TTF molecules in the model stack.

Shifts (Δx, Δy)/Å	Effective mass m*/me
(0.0, 0.0)	2.48
(0.0, 0.1)	2.55
(-0.1, 0.1)	2.57
(-0.25, 0.25)	3.28
(-0.5, 0.5)	4.70
(-1.25, 1.40)	12.62

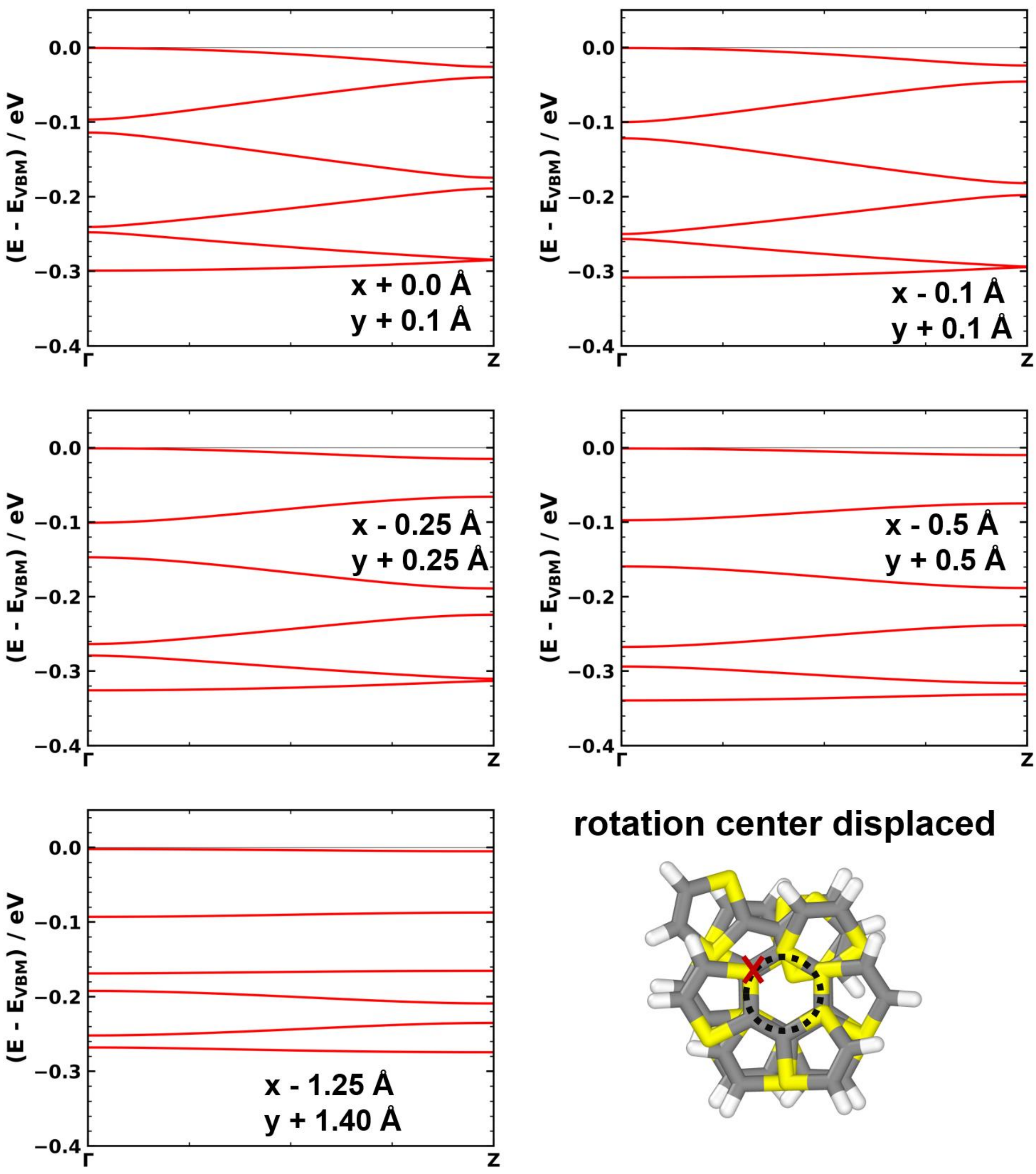

Figure S17. Electronic band structures for TTF model stacks where the rotation axis for one of the molecules is displaced from the equilibrium position.

4.2. Electronic band structures for the dimerization defect data

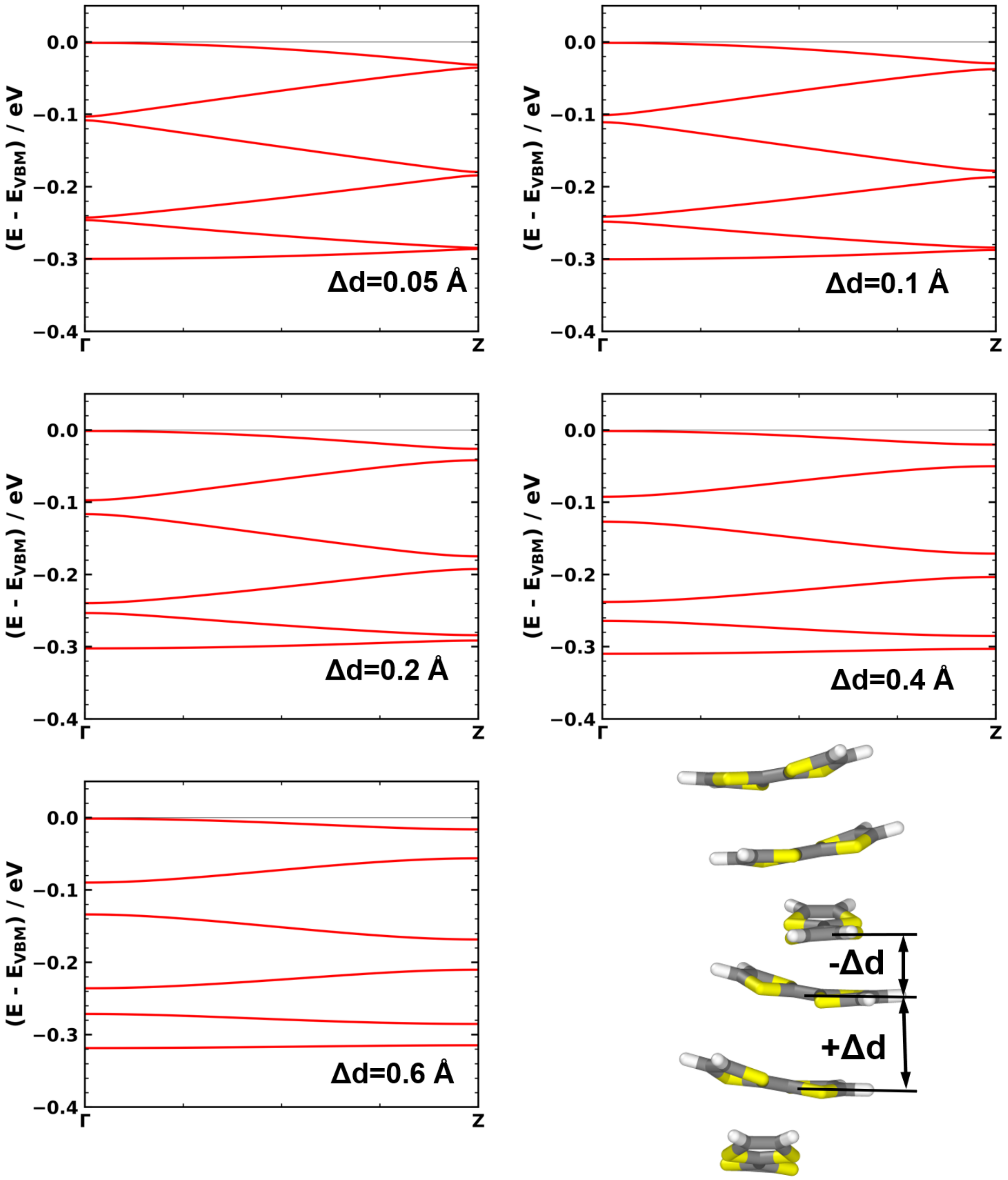

Figure S18. Electronic band structure of the valence band for the 6 repeat units TTF model stack with dimerization defects Δd.

4.3. Electronic band structures for the "displaced molecule" defect data

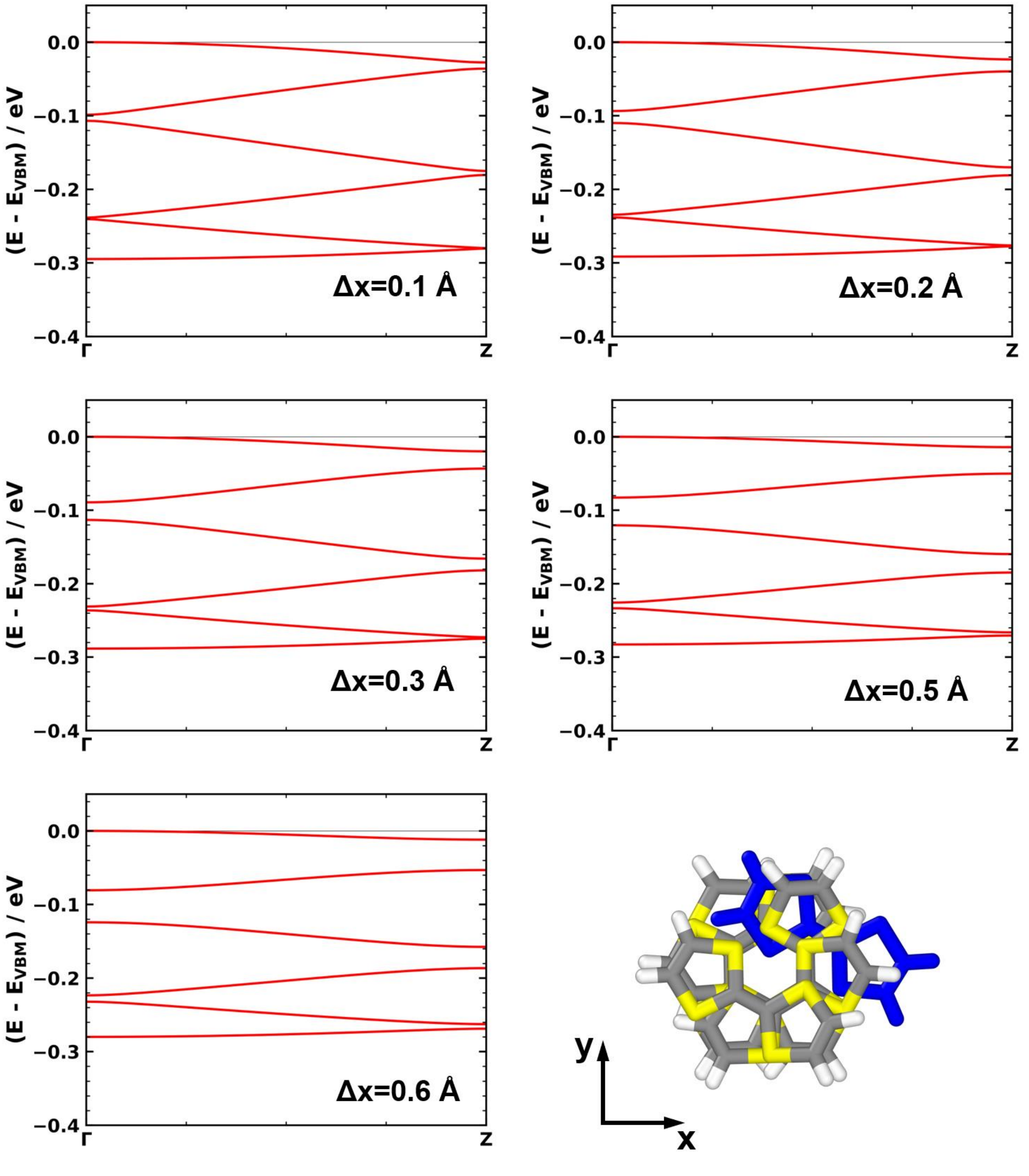

Figure S19. Electronic band structure of the valence band for the 6 repeat units TTF model stack with "displaced molecule" defects Δx.

References

1. Blum, V.; Gehrke, R.; Hanke, F.; Havu, P.; Havu, V.; Ren, X.; Reuter, K.; Scheffler, M. Ab initio molecular simulations with numeric atom-centered orbitals. *Comput. Phys. Commun.* **2009**, *180*, 2175–2196.
2. Winkler, C.; Mayer, F.; Zojer, E. Analyzing the Electronic Coupling in Molecular Crystals—The Instructive Case of α-Quinacridone. *Adv. Theory Simulations* **2019**, *2*, 1800204, doi:10.1002/adts.201800204.
3. Park, S.S.; Hontz, E.R.; Sun, L.; Hendon, C.H.; Walsh, A.; Van Voorhis, T.; Dincə, M. Cation-dependent intrinsic electrical conductivity in isostructural tetrathiafulvalene-based microporous metal-organic frameworks. *J. Am. Chem. Soc.* **2015**, *137*, 1774–1777, doi:10.1021/ja512437u.

4. Winkler, C.; Jeindl, A.; Mayer, F.; Hofmann, O.T.; Tonner, R.; Zojer, E. Understanding the Correlation between Electronic Coupling and Energetic Stability of Molecular Crystal Polymorphs: The Instructive Case of Quinacridone. *Chem. Mater.* **2019**, *31*, 7054–7069, doi:10.1021/acs.chemmater.9b01807.
